# Metabolic profiles in C_3_, C_3_–C_4_ intermediate, C_4_-like, and C_4_ species in the genus *Flaveria*

**DOI:** 10.1093/jxb/erab540

**Published:** 2021-12-15

**Authors:** Gian Luca Borghi, Stéphanie Arrivault, Manuela Günther, David Barbosa Medeiros, Emilia Dell’Aversana, Giovanna Marta Fusco, Petronia Carillo, Martha Ludwig, Alisdair R Fernie, John E Lunn, Mark Stitt

**Affiliations:** 1 Max Planck Institute of Molecular Plant Physiology, Am Mühlenberg 1, D-14476 Potsdam-Golm, Germany; 2 Universitá degli Studi della Campania, Dipartimento di Scienze e Tecnologie Ambientali, Biologiche e Farmaceutiche, Via Vivaldi 43, 81100 Caserta, Italy; 3 The University of Western Australia, School of Molecular Sciences, 35 Stirling Highway, 6009 Perth, Australia; 4 Bielefeld University, Germany

**Keywords:** C_4_ photosynthesis, Calvin–Benson cycle, evolution, *Flaveria*, metabolites, photorespiration

## Abstract

C_4_ photosynthesis concentrates CO_2_ around Rubisco in the bundle sheath, favouring carboxylation over oxygenation and decreasing photorespiration. This complex trait evolved independently in >60 angiosperm lineages. Its evolution can be investigated in genera such as *Flaveria* (Asteraceae) that contain species representing intermediate stages between C_3_ and C_4_ photosynthesis. Previous studies have indicated that the first major change in metabolism probably involved relocation of glycine decarboxylase and photorespiratory CO_2_ release to the bundle sheath and establishment of intercellular shuttles to maintain nitrogen stoichiometry. This was followed by selection for a CO_2_-concentrating cycle between phospho*enol*pyruvate carboxylase in the mesophyll and decarboxylases in the bundle sheath, and relocation of Rubisco to the latter. We have profiled 52 metabolites in nine *Flaveria* species and analysed ^13^CO_2_ labelling patterns for four species. Our results point to operation of multiple shuttles, including movement of aspartate in C_3_–C_4_ intermediates and a switch towards a malate/pyruvate shuttle in C_4_-like species. The malate/pyruvate shuttle increases from C_4_-like to complete C_4_ species, accompanied by a rise in ancillary organic acid pools. Our findings support current models and uncover further modifications of metabolism along the evolutionary path to C_4_ photosynthesis in the genus *Flaveria*.

## Teaser

Metabolite profiling and ^13^CO_2_labelling studies of *Flaveria*species on the spectrum between C_3_and C_4_photosynthesis reveal progressive re-wiring of photorespiration, nitrogen metabolism, carbon concentration shuttles, and the Calvin–Benson cycle.

## Introduction

In C_3_ photosynthesis, CO_2_ is assimilated by Rubisco in mesophyll cells. In a side reaction with O_2_, Rubisco catalysis produces 2-phosphoglycolate (2PG) that must be recycled at the expense of energy, CO_2_, and NH_3_ (Lorimer and Andrews, 1973; Bauwe *et al.*, 2010). C_4_ photosynthesis appeared 25–30 million years ago as an adaptation to a global climate change, which included a sharp decrease in atmospheric CO_2_ (Christin *et al.*, 2008; Zachos *et al.*, 2008), and has since then evolved independently in >60 angiosperm lineages (Sage *et al.*, 2011; Sage, 2016). In C_4_ photosynthesis, carbonic anhydrase (CA) converts CO_2_ into HCO_3_^–^ that is combined with phospho*enol*pyruvate (PEP) by PEP carboxylase (PEPC) to form oxaloacetate in the mesophyll. PEPC has a high HCO_3_^–^ affinity and no side reaction with O_2_. Oxaloacetate is transformed into more stable four-carbon (C_4_) metabolites, malate and aspartate (Asp), that diffuse from the mesophyll to the bundle sheath (BS) and are decarboxylated to generate a high CO_2_ concentration around Rubisco, which is located only in the BS. This high CO_2_ concentration suppresses the wasteful side reaction with O_2_. Three-C metabolites, such as pyruvate (Pyr) and alanine (Ala), move back to the mesophyll to regenerate the initial HCO_3_^−^ acceptor, PEP.

Under current atmospheric CO_2_ concentrations, C_4_ photosynthesis has several advantages over C_3_ photosynthesis (Sage *et al*., 2018); energetically wasteful photorespiration is decreased (Osmond and Harris, 1971; Sage *et al.*, 2012), water use efficiency is increased (Ghannoum, 2009), and nitrogen (N) use efficiency is improved (Ghannoum *et al.*, 2011). C_4_ species contain less Rubisco than C_3_ species because the high CO_2_ internal environment allowed evolution of lower specificity forms of Rubisco with a higher turnover number (*k*_cat_) (Brown, 1978; Schmitt and Edwards, 1981; Kubien *et al*., 2008; Kapralov *et al.*, 2011). These advantages have attracted great interest in engineering C_4_ photosynthesis into C_3_ crops (Matsuoka *et al.*, 2001; Häusler *et al.*, 2002; Miyao *et al.*, 2011; Ermakova *et al.*, 2019). Such crop improvement strategies may profit from understanding how C_4_ photosynthesis evolved.

C_4_ photosynthesis is a complex trait involving anatomical as well as biochemical differences from C_3_ photosynthesis. Almost all C_4_ species have a ‘Kranz’ anatomy with a ring of mesophyll cells surrounding photosynthetically active BS cells. Furthermore, compared with C_3_ species, C_4_ plants have a higher vein density, a large increase in PEPC expression in the mesophyll cells, loss of Rubisco and most of the rest of the Calvin–Benson cycle (CBC) components from mesophyll cells, and increased numbers of plasmodesmata between the BS and mesophyll cells (Muhaidat *et al.*, 2007; Lundgren *et al.*, 2014; Danila *et al.*, 2016, 2018). C_4_ photosynthesis is thought to have evolved in a stepwise manner, with pre-conditioning steps such as closer vein spacing occurring in a C_3_ background, followed by the acquisition of genetic regulatory elements, and the establishment of intermediary biochemical states (Langdale, 2011; Sage *et al.*, 2012; Mallmann *et al.*, 2014). Investigation of the evolution of C_4_ photosynthesis has been aided by genera including *Flaveria*, *Heliotropium*, *Salsola*, *Steinchisma*, and *Neurachne*, which contain species with intermediate modes of photosynthesis that are thought to represent steps on the evolutionary path from the ancestral C_3_ condition to a complete C_4_ syndrome (Ku *et al.*, 1983; Muhaidat *et al.*, 2011; Khoshravesh *et al.*, 2016, 2019;Schüssler *et al.*, 2017). Bayesian modelling indicated that different genera may have acquired the various anatomical, ultrastructural, and metabolic traits in different time sequences (Williams *et al.*, 2013). This flexibility may have facilitated convergent evolution of the complex C_4_ trait across so many lineages. Modelling also indicated that metabolic subtraits evolved as successive modules, with each module being associated with an increase in fitness defined as the amount of CO_2_ fixed by a given amount of Rubisco (Heckmann *et al.*, 2013; Heckmann, 2016).

Relocation of glycine (Gly) decarboxylation is thought to have been an initial metabolic step that increased the chance of C_4_ photosynthesis evolving. The glycine decarboxylase complex (GDC) is involved in photorespiration and converts two molecules of Gly into one molecule of serine (Ser) plus CO_2_ and NH_3_. In C_3_ plants, GDC is mainly located in mesophyll cells. Rawsthorne and colleagues noticed that *Flaveria* and *Moricandia* species with conspicuous Kranz anatomy had little or no GDC activity in their mesophyll cells and high GDC activity in their BS cells (Hylton *et al.*, 1988; Rawsthorne *et al.*, 1988*a*; Rawsthorne and Hylton, 1991), and predicted that they operate a photorespiration-driven carbon-concentrating mechanism (CCM). In this photorespiratory-based CCM (termed the C_2_ cycle, as Gly has two C atoms), Gly diffuses from the mesophyll to the BS cells, where it is decarboxylated, leading to a higher CO_2_ concentration within BS cells (Keerberg *et al.*, 2014), and Ser diffuses to the mesophyll. In agreement, expression of genes encoding proteins in photorespiration and the glutamine–oxoglutarate aminotransferase (GOGAT) pathway for NH_3_ assimilation in C_2_ species is as high as in C_3_ species and only falls in species that are considered to represent later steps in the evolution of C_4_ photosynthesis (Mallmann *et al.*, 2014). The C_2_ cycle improves recapture of photorespired CO_2_, lowers the photosynthetic CO_2_ compensation point, and would have been of selective advantage in a low CO_2_ world (Keerberg *et al.*, 2014; Lundgren, 2020).

Operation of the C_2_ cycle is thought to have created a context in which the next step towards C_4_ photosynthesis could occur (Monson *et al*., 1986; Bauwe and Kolukisaoglu, 2003; Sage, 2004; Bauwe *et al.*, 2010; Sage *et al.*, 2012; Busch *et al.*, 2013; Heckmann *et al.*, 2013; Schulze *et al.*, 2013; Williams *et al.*, 2013); consequently, species with a C_2_ cycle represent an evolutionarily significant subset among C_3_–C_4_ intermediates. In addition to modifications in the exchange of C between mesophyll and BS cells, the progressive evolution of a complete C_4_ syndrome involved changes to N stoichiometry that required large fluxes of organic acids and amino acids between mesophyll and BS cells (Monson and Rawsthorne, 2000). In a widely accepted version of the C_2_ cycle, two N atoms move (in two Gly molecules) into the BS but only one (in one Ser) moves back to the mesophyll, whilst the other is released as NH_3_ in the BS cells. Mallmann *et al.* (2014) employed flux balance analysis modelling to investigate the consequences of refixing NH_3_ in the BS cells. They predicted that N stoichiometry could be maintained by large-scale movement of 2-oxoglutarate (2OG) to the BS cells and glutamate (Glu) to the mesophyll or, if their movement is constrained, exchange of Pyr and Ala, or malate and Asp. N fluxes could also be balanced by allowing a C_4_-like cycle, with malate being synthesized by PEPC in the mesophyll and decarboxylated in the BS cells with Ala returning to the mesophyll cells. This analysis pointed to a possible causal relationship between the C_2_ cycle and the emergence of C_4_ photosynthesis.

C_3_ plants contain orthologues of genes encoding C_4_-related enzymes, transporters, and regulatory proteins (Aubry *et al.*, 2011) that were co-opted during the transition from C_3_–C_4_ to C_4_ photosynthesis (Sage, 2004; Mallmann *et al.*, 2014; Schlüter and Weber, 2016, 2020). Early C_4_ species are thought to have possessed a functional C_4_ cycle, but with non-optimal distribution of C_4_ enzymes and Rubisco between cell types. This is exemplified by some C_4_-like members of the genus *Flaveria*, including *F. palmeri*, *F. vaginata*, and *F. brownii* (Cheng *et al.*, 1988; Moore *et al.*, 1989), and *Sesuvium sesuvioides* (Bohley *et al.*, 2019).

The final step towards complete C_4_ status is thought to have included fine tuning of compartmentation and the kinetic and regulatory properties of enzymes (Sage, 2004; Heckmann *et al.*, 2013; Williams *et al.*, 2013). For example, compared with C_3_ species, Rubisco from C_4_ species generally has a lower CO_2_ affinity and a higher *V*_max_ (see above), and PEPC from C_4_ species is less sensitive to feedback inhibition by malate (Westhoff and Gowik, 2004; Gowik and Westhoff, 2011).

Current thinking about the evolution of C_4_ photosynthesis is based on studies of photosynthetic traits (e.g. CO_2_ fixation rates, CO_2_ compensation point, and water use efficiency), anatomy, gene expression, enzyme activities, and genome structure (reviewed in Schlüter and Weber, 2020). There have been studies of a small number of metabolites in a few species (Leegood and von Caemmerer, 1994; Gowik *et al.*, 2011; Arrivault *et al.*, 2019). However, we lack a comprehensive study of metabolite levels in multiple species on the evolutionary path between C_3_ and C_4_ photosynthesis. This study investigated the absolute contents of >50 primary metabolites in nine *Flaveria* species, representing all modes of photosynthesis found in this genus, and uses this information to test current ideas and provide new insights into the evolution of C_4_ photosynthesis.

## Materials and methods

### Materials

Solvents and chemicals were from Merck (https://www.merckmillipore.com) and Roche Applied Science (https://lifescience.roche.com/en_gb.html). N_2_, O_2_, and unlabelled CO_2_ were from Air Liquide (https://www.airliquide.com/), and ^13^CO_2_ (isotopic purity 99%) was from Campro Scientific GmbH (www.campro.eu). A commercial soil mix containing white peat, clay, coconut husk fibre, and Osmocote Start slow release fertilizer (1 g l^−1^) was obtained from Stender AG (Schermbeck, Germany).

### Plant material and harvest for metabolite measurements

Seeds of *Flaveria robusta* (C_3_ proto-Kranz), *F. anomala* (C_3_–C_4_ intermediate Type I), *F. ramosissima* (C_3_–C_4_ intermediate Type II), *F. palmeri* (C_4_-like), *F. brownii* (C_4_-like), *F. trinervia* (C_4_), and *F. bidentis* (C_4_), and cuttings of *F. vaginata* (C_4_-like) and *F. cronquistii* (C_3_) were kindly provided by Professor Peter Westhof (Heinrich Heine University, Düsseldorf, Germany). Cuttings of *F. vaginata* were directly rooted in soil in 30 cm plastic pots, and plants were grown through to seed production in a naturally illuminated polytunnel with the temperature maintained above a minimum of 10 °C. Seeds were germinated on moist soil in a controlled-environment chamber with a 16 h day (20 °C)/8 h night (18 °C) cycle, 150 µmol m^−2^ s^−1^ irradiance, and relative humidity (RH) of 70%. After 2 weeks, individual seedlings were transferred to 6 cm diameter plastic pots containing soil, repotted into 18 cm diameter plastic pots 1 week later, and into 30 cm diameter plastic pots 2 weeks before harvest. All growth was under a 16 h day (26 °C)/8 h night (22 °C) cycle, average irradiance 350 µmol m^−2^ s^−1^, and 70% RH. Due to seed sterility, *F. cronquistii* was propagated from sterile cuttings [sterilization medium: 0.5% (w/v) Murashige and Skoog medium containing 5% (w/v) Plant Preservation Medium (PPM^®^; Plant Cell Technologies Inc.)] grown initially in tissue culture [growth medium: 1% (w/v) Murashige and Skoog medium, 6.8% (w/v) agarose, 1% (w/v) PPM^®^] for 10 d before transferring well-rooted cuttings to 10 cm diameter plastic pots containing soil. After 2 weeks under a 16 h day (20 °C)/8 h night (18 °C) cycle, 150 µmol m^−2^ s^−1^, and 70% RH, plantlets were repotted into 18 cm diameter plastic pots, moved to a 16 h day (26 °C)/8 h night (22 °C) cycle, 350 µmol m^−2^ s^−1^ irradiance, and 70% RH, and repotted in 30 cm diameter plastic pots 2 weeks before harvest.

Leaves of most species were harvested 55–56 d after sowing, the slower growing *F. ramosissima* and *F. trinervia* 79–80 d after sowing, and *F. cronquistii* 55 d after transfer to soil. First fully expanded leaves were removed under an irradiance of 350 µmol m^−2^ s^−1^ and quickly (<2 s) plunged in a bath of liquid N_2_ without shading or turning. Multiple leaves from four different plants (four biological replicates), were harvested (Supplementary Table S1).

### Plant material and harvest for ^13^CO_2_ labelling

Four species from Clade A (Supplementary Fig. S1; McKown *et al.*, 2005; Lyu *et al.*, 2015) were chosen to represent different photosynthesis modes: *F. robusta* (C_3_), *F. ramosissima* (C_3_–C_4_), *F. palmeri* (C_4_-like), and *F. bidentis* (C_4_). Seeds were germinated on moist soil (as above) under a 16 h day (20 °C)/8 h night (18 °C) cycle, 150 µmol m^−2^ s^−1^, and 70% RH. After 3 weeks, individual seedlings were transferred into 10 cm diameter plastic pots and grown under a 16 h day (26 °C)/8 h night (22 °C) cycle, average irradiance 500 µmol m^−2^ s^−1^, and 70% RH, until labelling 49/50 d after sowing. Labelling was conducted by placing a single leaf per plant (i.e. a biological replicate) in a custom-made transparent gas-tight acrylic chamber connected to a gas flowmeter, infrared CO_2_ analyser (LI-800 Gashound; LI-COR Biosciences), humidifier bottle, and gas bottles (Ermakova *et al.*, 2020). Irradiance at leaf level within the chamber was 500 µmol m^−2^ s^−1^. After 2 min acclimation with 420 µl l^−1^ CO_2_, 21% O_2_, 78% N_2_, the gas flow was switched to 420 ppm ^13^CO_2_, 21% O_2_, 78% N_2_ for 40 min or 60 min before harvest. Metabolism was quenched by rapidly flooding the chamber with liquid N_2_ without opening the chamber and avoiding any shading of the leaf (Ermakova *et al.*, 2020; Supplementary Table S1). Unlabelled (time zero) control samples were quenched with liquid N_2_ at the end of the acclimation phase without introducing ^13^CO_2_ into the labelling chamber.

### Metabolite analyses

Samples were ground to a fine powder at liquid N_2_ temperature using a ball mill (Retsch GmbH) at 25 Hz speed and stored at –80 °C until analysis. Pyruvate, PEP, 3-phosphoglycerate (3PGA), and ATP were measured enzymatically in trichloracetic acid extracts from 50 mg FW aliquots of tissue (Merlo *et al.*, 1993). Other organic acids, sugar phosphates, and cofactors were measured, after methanol/chloroform extraction from 15 mg FW aliquots, using reverse phase HPLC coupled with a tandem MS (LC-MS/MS) platform (Arrivault *et al.*, 2009). Stable isotopically labelled internal standards were added to correct for matrix effects for a subset of metabolites (Arrivault *et al.*, 2015). Amino acids were measured by HPLC on 20 mg FW aliquots after ethanolic extraction and *o*-phthalaldehyde derivatization (Carillo *et al.*, 2005). Supplementary Table S2 provides a list of metabolites, abbreviations, and analytical methods.

### ^13^C enrichment analyses

^13^CO_2_-labelled samples were ground as above and extracted for GC-time-of-flight (TOF)-MS as in Lisec *et al.* (2006) and for LC-MS/MS as in Arrivault *et al.* (2009). Peaks obtained from GC-TOF-MS analyses were assigned to metabolites by comparing mass spectra and GC retention times with database entries and those of authentic standards available in a reference library from the Golm Metabolome Database (Kopka *et al.*, 2005). Chromatograms obtained from LC-MS/MS were analysed as in Arrivault *et al.* (2017). Natural abundance was corrected using the CORRECTOR software (www.mpimp-golm.mpg.de/719693/Bioinformatik-Tools; Huege *et al.*, 2014).

### Statistical analyses

Clustering and principal component (PC) analyses were performed in R Studio version 1.2.5033 (www.rstudio.com) integrated with R version 3.6.1 (www.r-project.org/). One-way ANOVAs with Holm–Sidak post-hoc tests were computed using SigmaPlot vers. 14.0 (Systat Software, Inc.).

## Results

### Global analysis of metabolite levels in nine *Flaveria* species

The *Flaveria* genus includes two basal C_3_ species and two phylogenetically separated clades, with Clade A containing C_3_–C_4_ intermediate, C_4_-like, and C_4_ species, and Clade B containing C_3_–C_4_ intermediate and a C_4_-like species (Supplementary Fig. S1; McKown *et al.*, 2005; Lyu *et al.*, 2015). For our analyses, nine species were selected, comprising the two basal C_3_ species (*F. robusta* and *F. cronquistii*), two C_3_–C_4_ intermediates (*F. ramosissima* and *F. anomala*; from Clades A and B, respectively), three C_4_-like species (*F. palmeri*, *F. vaginata*, and *F. brownii*; Clades A, A, and B, respectively), and two complete C_4_ species (*F. bidentis* and *F. trinervia*; both Clade A). After establishment, all species were grown in the same controlled conditions. The first fully expanded leaves were harvested by flash-freezing under growth irradiance. We measured 52 metabolites, encompassing sugar phosphates, organic acids, and amino acids, and including most of the metabolites in the CBC, C_4_ photosynthesis (from here onward termed ‘CCM-related’ metabolites), photorespiration, the tricarboxylic acid (TCA) cycle, and sucrose and starch synthesis (Supplementary Table S2). Absolute metabolite contents are provided in Supplementary Dataset S1. The data for CBC metabolites and 2PG levels in the two complete C_4_ species *F. bidentis* and *F. trinervia* were previously published in Arrivault *et al.* (2019).

**Fig. 1.**
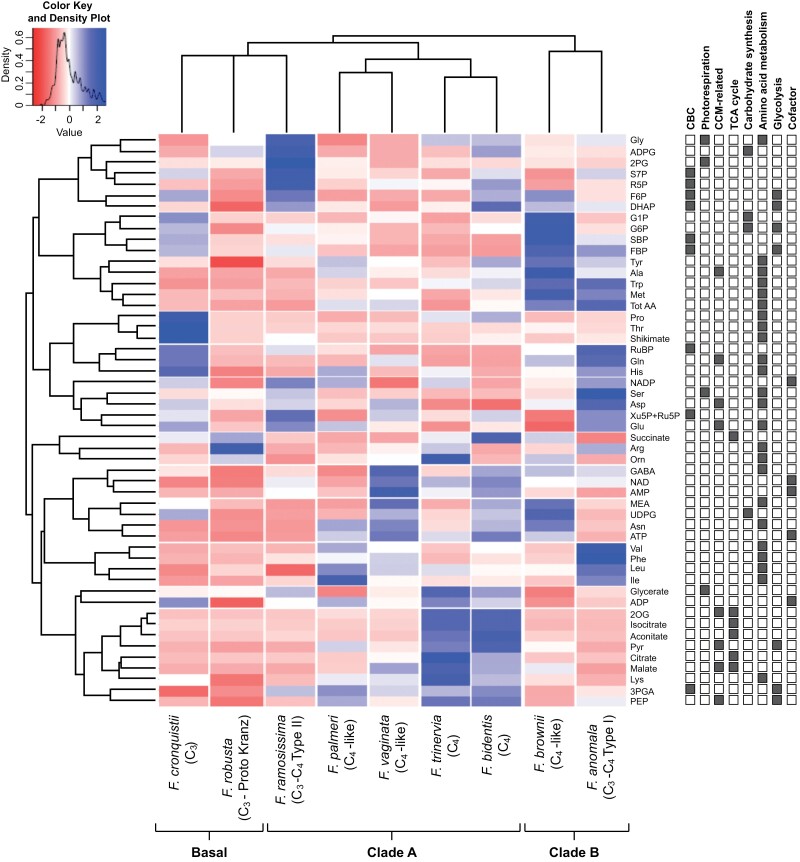
Heatmap and dendrograms of absolute metabolite levels in nine *Flaveria* species. The heatmap was generated by two-way clustering: each column represents a *Flaveria* species, and each row represents a metabolite. The *Flaveria* species is indicated at the bottom of each column, as well as its photosynthesis mode and taxonomical grouping according to current knowledge. Species (top) and metabolite (left-hand side) dendrograms were calculated using *Z*-scored data. The data were Z-scored by expressing the average level of a metabolite for (*n*=3–4; each replicate obtained by harvesting several newly fully expanded leaves from one *Flaveria* plant) in a given species as a fraction of the average across all species. The heatmap cell colour intensity represents the average *Z*-score for the metabolite in that species. The colour key and data distribution plot for the heatmap are shown in the insert in the upper-left corner. The right-hand subpanel indicates the sector of metabolism in which a given metabolite is involved; metabolic sectors are indicated at the top of the subpanel, and the involvement of a metabolite by a grey box. In some cases, a metabolite is assigned to more than one sector. This aspect of the data is explored further in Fig. 4. For metabolite abbreviations, refer to Supplementary Table S2, and for the original data see Supplementary Dataset S1.

**Fig. 2.**
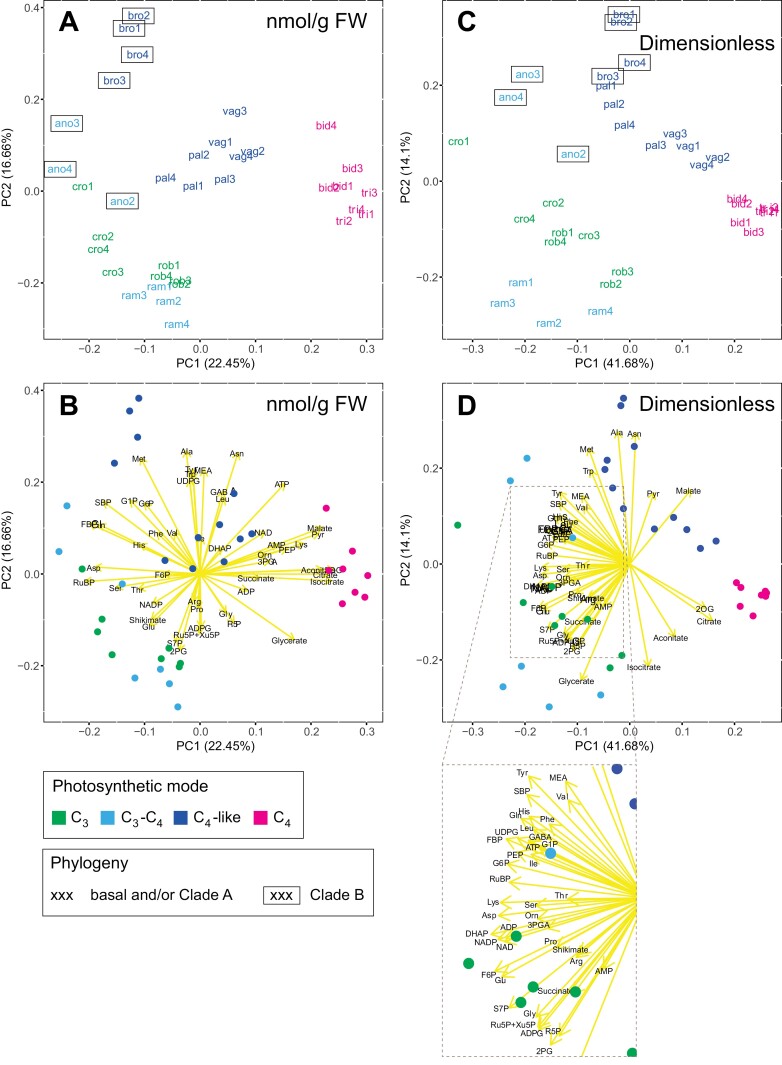
Principal component (PC) analysis of metabolite profiles in nine *Flaveria* species. (A, B) PC analysis of the FW-normalized dataset. (C, D) PC analysis using a dimensionless dataset. (A, C) Distribution of samples along the two first two PCs, with each sample being represented by a coloured label indicating the species and biological replicate number. (B, D) Metabolite eigenvectors driving sample separation are shown in yellow, while individual samples appear as coloured dots. In all panels, the colour code represents the different photosynthetic modes, as indicated by the key below the figure. In (A) and (C), a box denotes Clade B species, as indicated by the key below the figure. Species abbreviations are, alphabetically: ano, *F. anomala* (Clade B, C_3_–C_4_); bid, *F. bidentis* (Clade A, C_4_); bro, *F. brownii* (Clade B, C_4_-like); cro, *F. cronquistii* (basal, C_3_); pal, *F. palmeri* (Clade A, C_4_-like); ram, *F. ramosissima* (Clade A, C_3_–C_4_); rob, *F. robusta* (basal, C_3_); tri, *F. trinervia* (Clade A, C_4_); vag, *F. vaginata* (Clade A, C_4_-like). For metabolite abbreviations, refer to Supplementary Table S2, and for the original data see Supplementary Dataset S1.

To provide a first overview, metabolite contents (per unit FW) were *Z*-score normalized and used in two-way clustering (Fig. 1; Supplementary Dataset S1) and PC analysis (Fig. 2A, B; Supplementary Dataset S1). PC analysis was also performed on data that were transformed to express each metabolite as the amount of C in that metabolite normalized to total C in all measured metabolites, termed a ‘dimensionless’ dataset (Fig. 2C, D; Supplementary Dataset S1; Arrivault *et al.*, 2019; Borghi *et al.*, 2019). This normalization removes possible bias due to differences in leaf protein and/or water content. These global analyses separated species based on photosynthetic mode and their phylogeny.

The clustering analysis is shown as a heatmap in Fig. 1. The basal C_3_ species *F. cronquistii* and *F. robusta* grouped with the C_3_–C_4_*F. ramosissima* of Clade A in a polytomy. The C_4_-like species *F. palmeri* and *F. vaginata* from Clade A formed a sister group to that composed of the two Clade A C_4_ species, *F. bidentis* and *F. trinervia*, used in the study. This group of C_4_-like and complete C_4_ species are sister to the basal C_3_*F. ramosissima* cluster, while the Clade B C_4_-like *F. brownii* and C_3_–C_4_*F. anomola* formed a distinct cluster.

In the PC analysis, PC1 and PC2 captured 22.45% and 16.66%, respectively, of the total variance in the FW-normalized dataset (Fig. 2A, B; Supplementary Table S3), and 41.68% and 14.1%, respectively, of the total variance in the dimensionless dataset (Fig. 2C, D; Supplementary Table S3). Both datasets separated species based on mode of photosynthesis and on phylogeny. For Clade A species, the two complete C_4_ species (*F. bidentis* and *F. trinervia*) were clearly separated from the two C_4_-like species (*F. vaginata* and *F. palmeri*) and the C_3_–C_4_ species (*F. ramosissima*), as well as from the basal C_3_ species *F. cronquistii* and *F. robusta*. This separation was found in PC1 and PC2 (Fig. 2A, C). Compared with the basal C_3_ species, the C_3_–C_4_*F. ramosissima* was slightly displaced in a negative direction in PC2, while the two C_4_-like species (*F. vaginata* and *F. palmeri*) were strongly displaced in a positive direction. This suggests that the evolutionary path from C_3_ through C_3_–C_4_ and C_4_-like to complete C_4_ may not involve a progressive change in metabolite levels, but instead the establishment of discrete states at each stage in the evolutionary process, especially the C_3_–C_4_ stage. For Clade B species, C_3_–C_4_*F. anomala* was largely separated from the basal C_3_ species, again mainly in PC2, but, unlike *F. ramosissima*, in a positive direction, lying quite close to the Clade B C_4_-like species *F. brownii* (Fig. 2A, C). In the analysis with FW-normalized data (Fig. 2A, B), PC1 and PC2 captured 39.11% of the total variance. We extended the analysis to include PC3 (12.52%) and PC4 (11.34%) (Supplementary Fig. S2), which, together with PC1 and PC2, explained >60% of the total variance (Supplementary Table S3). In PC3 there was no clear pattern. PC4 separated C_3_ species from C_3_–C_4_*F. anomola* and *F. ramosissima*, but not entirely from C_4_-like and C_4_ species (Supplementary Fig. S2).

We next examined which metabolites drove the separations in the clustering and PC analyses. The separation of the C_3_ species from other species, except Clade A C_3_–C_4_*F. ramosissima* (see below), was driven by 2PG, Gly, Ser, and glycerate, several CBC intermediates (e.g. RuBP, Ru5P+Xu5P, R5P, and S7P), some amino acids (Pro, Thr, His, Gln, Arg, and Glu), and the aromatic amino acid precursor shikimate (Figs 1, 2B, D). *Flaveria ramosissima*, which is an advanced C_3_–C_4_ species in Clade A, lays close to the basal C_3_ species, resembling the hierarchical clustering shown in Fig. 1. Its separation from the other species was due to comparatively high levels of some CBC (S7P, F6P, and R5P) and photorespiratory (2PG, Gly, and glycerate) metabolites (Figs 1, 2B, D). Separation of the two Clade A C_4_-like species from the C_3_–C_4_ intermediates and C_3_ species occurred mainly in PC1 and was driven by higher levels of CCM-related metabolites (malate and Pyr) and some amino acids (Ala and Asn). High levels of TCA and other respiratory intermediates such as 2OG, citrate, isocitrate. and aconitate and, to a lesser extent, Pyr and malate (only clear in the analysis with FW-normalized data), were largely responsible for the separation of complete C_4_ species from C_4_-like species. The separation of *F. anomala*, an early branching C_3_–C_4_ species in Clade B, from basal C_3_ species was driven by high Ser, SBP, and FBP, and a distinctive amino acid profile (high Ala, Asn, Met, Trp, Tyr, and Gln), a pattern that was also largely responsible for the placement of the Clade B C_4_-like *F. brownii* in the PC analyses.

In summary, species in Clade B differ from those in Clade A in having higher levels of some amino acids and sugar phosphates. Compared with basal C_3_ species, the Clade A C_3_–C_4_*F. ramosissima* tends to have higher CBC and photorespiratory metabolites; C_4_-like species, regardless of clade, have higher CCM-related intermediates; and the change from C_4_-like to a complete C_4_ syndrome is associated with higher levels of other organic acids (Figs 1, 2B, D). Overall, changes between C_3_ and C_3_–C_4_ species are captured mainly in PC2 and are smaller and partly orthogonal to the changes presumably associated with subsequent evolutionary steps to C_4_-like and complete C_4_ species that are captured mainly in PC1. This is especially so for Clade A species.

### Changes of individual metabolites between species

**Fig. 3.**
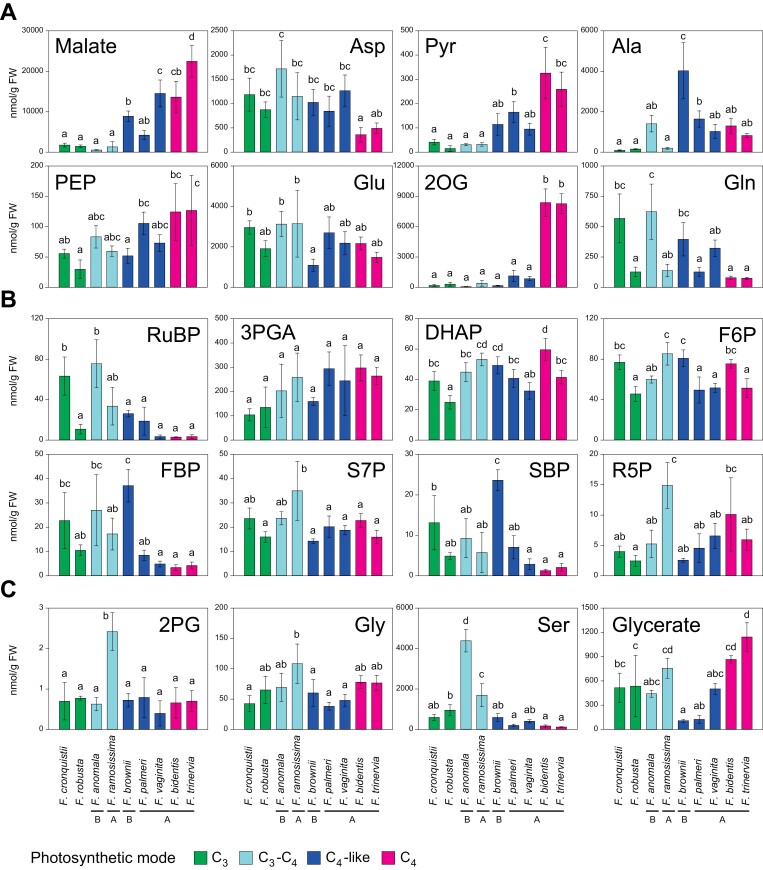
Absolute amounts of metabolites in nine *Flaveria* species. (A) CCM-related metabolites, (B) selected CBC metabolites, and (C) photorespiratory metabolites. The colour of each bar represents the different photosynthetic modes, as indicated by the key below the figure. In the sets of C_3_–C_4_ and C_4_-like species, the Clade B species (*F. anomala* and *F. brownii,* respectively) are placed to the left of the Clade A species; clade is also indicated as ‘A’ or ‘B’ below the species name. The amounts are plotted as average (nmol g^–1^ FW) ±SD (*n*=3–4). Letters above each bar represent post-hoc pairwise comparison grouping (Holm–Sidak method, *P*<0.05). For metabolite abbreviations. refer to Supplementary Table S2, and for the original data see Supplementary Dataset S1. The data in (B) for CBC metabolite levels in *F. bidentis* and *F. trinervia* were included in a previous publication (Arrivault *et al.*, 2019).

To investigate responses that might be masked in the above global analyses, the responses of individual metabolites were examined (Fig. 3; Supplementary Fig. S3)

Figure 3A shows amounts of malate, Asp, Pyr, Ala, and PEP which are directly involved in the C_4_ CCM, and levels of Glu, 2OG, and Gln, which potentially facilitate aminotransferase reactions in C_4_ photosynthesis and/or could be involved in shuttles that return amino groups from the BS to the mesophyll in C_3_–C_4_ photosynthesis. These metabolites were relatively low in both C_3_ species, except for Asp and Glu, whose levels resembled those in most other *Flaveria* species. The only major difference between the two C_3_ species was for Gln, which was significantly higher in *F. cronquistii* than in *F. robusta*. There were some noticeable differences between the two C_3_–C_4_ species, with Gln being 4-fold being higher, and Ala 5-fold lower in *F. anomala* than in *F. ramosissima*. Comparison of all four species indicated that the transition from C_3_ to C_3_–C_4_ was not accompanied by consistent and significant increases in the content of any CCM-related metabolites. The transition from C_3_–C_4_ to a C_4_-like state was accompanied by an increase (significant in some pairwise comparisons) in the contents of malate, Pyr, and Ala. That said, the three C_4_-like species were quite diverse: for example, they had differing levels of malate, Ala, PEP, and Gln. These differences might be due to differing evolutionary histories, reflected in *F. brownii* (high Ala, low PEP, low Glu, and high Gln) belonging to Clade B, while the two other C_4_-like species are in Clade A. In the transition from C_4_-like to a complete C_4_ syndrome, there was a trend towards even higher malate, Pyr, and PEP levels, no consistent change in the amounts of Ala and Glu, a decrease in Asp and Gln, and an increase in 2OG and other TCA cycle intermediates such as aconitate, citrate, and isocitrate (Supplementary Fig. S3).

CBC metabolite levels were less dependent on photosynthesis mode (Fig. 3B). Comparing the two C_3_ species, RuBP, DHAP, and F6P were significantly higher in *F. cronquistii* than in *F. robusta*, whereas no significant differences were found for 3PGA, FBP, S7P, SBP, and R5P. It might be noted that some of these metabolites (e.g. DHAP and F6P) are also involved in sucrose synthesis. Relative to the two C_3_ species, the C_3_–C_4_ species (*F. anomala* and *F. ramosissima*) showed few significant or consistent changes in CBC metabolite levels. Both C_3_–C_4_ species had significantly higher RuBP, DHAP, F6P, and S7P compared with *F. robusta*, but not with *F. cronquistii*. Compared with C_3_–C_4_ species, C_4_-like species showed a trend towards lower RuBP (significant in the comparison with *F. anomala* but not with *F. ramosissima*). The Clade B species *F. brownii* was the most divergent of the three C_4_-like species, with significantly higher FBP, F6P, and SBP than the Clade A C_4_-like species. Complete C_4_ species showed a trend (significant for some metabolites and pairwise comparisons) towards lower RuBP, FBP, and SBP than in Clade A C_4_-like species. Moving from C_3_–C_4_ to C_4_-like to complete C_4_ species, there was a consistent trend towards lower amounts of RuBP, FBP, and SBP, but not of other CBC intermediates.

Figure 3C shows contents of photorespiratory intermediates. No significant differences were detected between the two C_3_ species (*F. cronquistii* and *F. robusta*). Both C_3_–C_4_ intermediates showed significant increases in Ser compared with the C_3_ species. No consistent differences were found for 2PG and Gly; neither of these metabolites increased in *F. anomala* relative to the C_3_ species, while *F. ramosissima* had significantly higher amounts of 2PG compared with *F. cronquistii* and *F. robusta*, and significantly higher amounts of Gly compared with *F. robusta*. It is noteworthy that Ser levels are far higher than Gly levels (15- to 50-fold) in the C_3_–C_4_ species. Compared with C_3_–C_4_ intermediates, C_4_-like *Flaveria* species showed lower levels of Gly (significant in some pairwise comparisons), Ser (significant in all pairwise comparisons), and glycerate (significant in some pairwise comparisons). Compared with C_4_-like species, complete C_4_ species had similar levels of 2PG, consistently but non-significantly higher Gly and lower Ser, and higher glycerate (significant in some pairwise comparisons). Maybe unexpectedly, the complete C_4_ species had the highest glycerate levels of all nine species.

### Changes of classes of metabolites between species

**Fig. 4.**
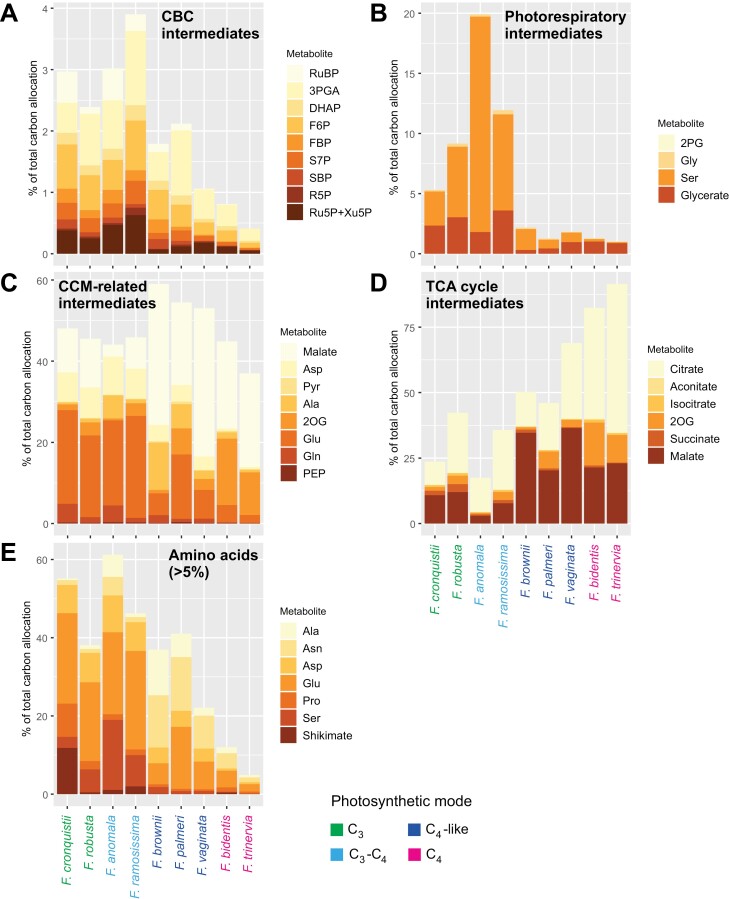
Carbon allocation to different sectors of metabolism in nine *Flaveria* species. C allocation was calculated using a dimensionless dataset. The percentage of total C in a given metabolite is depicted in the stacked bar diagrams as the average (*n*=3–4); metabolites are ordered according to their sequence in the metabolic pathway or sector. A metabolite can appear in multiple panels. The colour assigned to each metabolite is indicated in the insert of each panel: (A) CBC intermediates, (B) photorespiratory intermediates, (C) CCM-related intermediates, (D) TCA cycle intermediates, and (E) amino acids (only amino acids containing >5% of the total C pool). The colour of each species’ name represents the corresponding photosynthetic mode as indicated by the key at the bottom right. For metabolite abbreviations, refer to Supplementary Table S2, and for the original data see Supplementary Dataset S1.

The above analyses of the levels of individual metabolites revealed some significant differences, but also indicated that others did not show consistent differences across the nine *Flaveria* species (Fig. 3; Supplementary Fig. S3). The results shown in Figs 1 and 3 indicated that changes in metabolite levels may be focused in particular sectors of metabolism. This possibility was explored further using the ‘dimensionless’ dataset (Fig. 4; Supplementary Dataset S1). Metabolites were grouped based on pathways or sectors of metabolism: CBC, photorespiratory, CCM-related, TCA cycle intermediates, and amino acid. The class ‘CCM-related’ contained not only metabolites that participate in intercellular shuttles in C_4_ photosynthesis, but also metabolites potentially involved in shuttles in C_3_–C_4_ species. Some metabolites were assigned to more than one class (e.g. malate and 2OG were assigned to ‘CCM-related’ and ‘TCA cycle’). The analysis shown in Fig. 4 revealed that the evolution of C_4_ photosynthesis is accompanied by major changes in C allocation between sectors of metabolism.

Metabolites in the CBC accounted for only a small fraction of the C in central metabolism (Fig. 4A), reflecting their small pool sizes and rapid turnover (Szecowka *et al.*, 2013; Ma *et al.*, 2014; Arrivault *et al.*, 2017). They accounted for 2.4–3% of total C in C_3_ species and 3.0–3.8% in C_3_–C_4_ species, falling to 1.1–2.0% in C_4_-like species and to 0.4–0.8% in complete C_4_ species. Photorespiratory metabolites accounted for 5–10% of total C in central metabolism in *Flaveria* species using C_3_ photosynthesis (Fig. 4B). This reflects the large pool sizes of Gly, Ser, and glycerate in C_3_ species (Szecowka *et al.*, 2013; Ma *et al.*, 2014). C allocation to photorespiratory metabolites rose to 13–20% in C_3_–C_4_ species, and fell to <2.5% in C_4_-like and complete C_4_ species. This was mainly due to changes in Ser. Metabolites assigned to the CCM-related category accounted for 44–47% of all C in central metabolism in C_3_ and C_3_–C_4_ species, slightly more (53–58%) in C_4_-like species, and slightly less (38–44%) in complete C_4_ species (Fig. 4C). It should be noted that in any given species, some of these metabolites may not be involved in intercellular shuttles and, when they are, only part of the total pool may be involved (see Introduction and below). C allocation to 2OG was highest in complete C_4_ species. Ala showed a unique pattern, being relatively high in both Clade B species (5–15%) and the C_4_-like Clade A species, *F. palmeri* (just below 5%). TCA cycle intermediates accounted for 20–45% of total C in central metabolism in C_3_ species and C_3_–C_4_ species, 45–70% in C_4_-like species, and >75% in complete C_4_ species (Fig. 4D). In addition to malate and 2OG, the TCA cycle metabolite showing the greatest difference between species was citrate, which accounted for >30% of total C in C_4_-like *F. vaginata* and the complete C_4_ species. Amino acids that contributed >5% of total C in at least one species were also examined (Fig. 4E). Amino acids showed an opposite trend to TCA cycle metabolites, accounting for 40–50% of total C in C_3_ species, 45–60% in C_3_–C_4_ species, 20–40% in C_4_-like species, but only 5–10% in complete C_4_ species.

### ^13^CO_2_ labelling experiments in four selected *Flaveria* species

As many metabolites are compartmented, with large pools in vacuoles or non-photosynthetic cells, total content may not provide accurate information about the size of the pool that is directly involved in photosynthesis (Szecowka *et al.*, 2013; Arrivault *et al.*, 2017). One way to investigate compartmentation is by ^13^CO_2_ labelling. Experiments in C_3_ tobacco (Hasunuma *et al.*, 2010), C_3_ Arabidopsis (Szecowka *et al.*, 2013; Ma *et al.*, 2014), C_3_ cassava (Arrivault *et al.*, 2019), and the C_4_ plant maize (Weissmann *et al.*, 2016; Arrivault *et al.*, 2017) have shown that CBC metabolites label to high enrichment within minutes. Enrichment in photorespiratory metabolites rises more slowly, but usually plateaus by 40 min. In maize, labelling of metabolites involved in the CCM plateaus by 15–20 min. For organic acids and amino acids, in particular, enrichment can plateau at quite a low value. The labelled portion is thought usually to reflect the pool that is actively involved in photosynthesis, namely the pool that is directly downstream of C assimilation, although in some cases incomplete labelling can result from entry of unlabelled C from downstream metabolites (e.g. starch or photorespiratory intermediates) into a single pool that is directly involved in photosynthesis (Sharkey *et al.*, 2020).

Four *Flaveria* species were chosen for ^13^CO_2_ labelling experiments; a basal C_3_ species (*F. robusta*), and three species from Clade A, a C_3_–C_4_ (*F. ramosissima*), a C_4_-like (*F. vaginata*), and a complete C_4_ species (*F. bidentis*). We focused on Clade A to cover the spectrum of photosynthetic types, including complete C_4_, in species with a more recent shared evolutionary history, thereby minimizing any differences that reflect phylogeny rather than photosynthetic type. Pulse durations of 40 min or 60 min were chosen, with the expectation that enrichment would be at or close to a plateau (see above). Enrichment was determined for two metabolites in the CBC and 10 metabolites in CCM or photorespiratory pathways (highlighted in red in Supplementary Table S2; data are provided in Supplementary Dataset S2).

Supplementary Fig. S4 shows the differences in ^13^C enrichment in the four *Flaveria* species. Although the estimates of enrichment are approximate, the values for the C_3_ and C_4_*Flaveria* species generally resemble those reported in extensive studies of other C_3_ and C_4_ species (Hasunuma *et al.*, 2010; Szecowka *et al.*, 2013, Ma *et al.*, 2014; Weissmann *et al.*, 2016; Arrivault *et al*., 2017, 2019). Enrichment in the CBC intermediates 3PGA and DHAP was high (>80%) in all four species (Supplementary Fig. S4A, B). Enrichment was also high in PEP, which is important as it means that low enrichment in any of the downstream CCM-related metabolites provides evidence for slow synthesis and/or the presence of compartmented pools. Enrichment in malate was weak (<10%) in the C_3_ species *F. robusta*, only marginally higher in the complete C_4_ species *F. bidentis* and the C_3_–C_4_*F. ramosissima*, and slightly higher (~25%) in the C_4_-like *F. palmeri*. This resembles previous studies of malate in C_3_ and C_4_ species (Hasunuma *et al.*, 2010; Szecowka *et al.*, 2013; Ma *et al.*, 2014; Weissmann *et al.*, 2016; Arrivault *et al.*, 2017, 2019). Enrichment in Asp was low (~20%) in the C_3_*Flaveria* species, and higher in the C_3_–C_4_, C_4_-like, and C_4_ species (~60, 85, and 75%, respectively), which all lay in the range previously reported for maize (Weissmann *et al.*, 2016; Arrivault *et al.*, 2017). Enrichment in Pyr was quite low (~30%) in the C_3_*Flaveria* species (similar to Arabidopsis, see Szecowka *et al*., 2013) and C_3_–C_4_*Flaveria* species, and higher (>50%) in the C_4_-like and complete C_4_*Flaveria* species (similar to maize; Weissmann *et al.*, 2016; Arrivault *et al.*, 2017). Enrichment in Ala was ~50% in the C_3_*Flaveria* species, 60% in the C_3_–C_4_ species, and 70–80% in the C_4_-like and complete C_4_ species. Overall, these analyses of enrichment point to increased C flow from the CBC to Asp in C_3_–C_4_, C_4_-like, and C_4_ species, and to Pyr and Ala in C_4_-like and C_4_ species. Glu and 2OG were weakly and incompletely labelled in all four *Flaveria* species, resembling previous studies of other C_3_ and C_4_ species (Szecowka *et al.*, 2013; Ma *et al.*, 2014; Ishihara *et al.*, 2015; Arrivault *et al.*, 2017, 2019). For photorespiratory metabolites, enrichment was high in Gly (75–90%) and Ser (70–80%) in all four *Flaveria* species, as found previously for other C_3_ and C_4_ species (Szecowka *et al.*, 2013; Ma *et al.*, 2014; Ishihara *et al.*, 2015; Arrivault *et al.*, 2017, 2019). Enrichment in glycerate was comparatively low (~20%) in the C_3_*Flaveria* species, and higher in the C_3_–C_4_ (>60%), C_4_-like, and complete C_4_ species (~50%).

### Estimation of active pools in *Flaveria* species, and comparison with maize

**Fig. 5.**
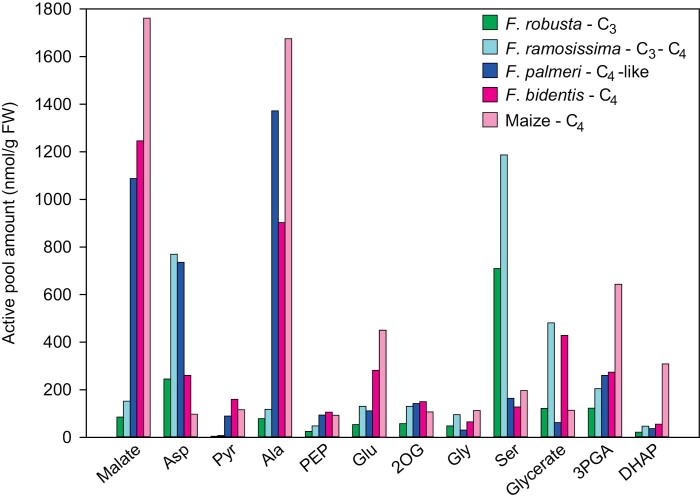
Estimated active pool of metabolites in four *Flaveria* species and maize. Amounts (nmol g^–1^ FW) are shown for metabolites involved in the CCM, the photorespiratory pathway, and two metabolites from the CBC. Details for calculations are presented in Supplementary Table S4. Estimates for maize are from Arrivault *et al*. (2017) and additional 3PGA quantification. For metabolite abbreviations, refer to Supplementary Table S2.

To estimate the active pool of each metabolite in each of the four *Flaveria* species, total metabolite content was multiplied by ^13^C enrichment (Fig. 5; Supplementary Table S4). For comparison, Fig. 5 also shows active pools for maize, estimated from published data (Arrivault *et al.*, 2017). In cases where a clear labelling plateau was not reached, the estimated active pool is a minimum value. The estimates highlight differences in active pool size between the four *Flaveria* species, revealing at what stage in the C_3_ to C_4_ evolutionary process major changes in photosynthetic metabolism occurred and indicating the extent to which the complete C_4_*Flaveria* species resembles maize.

The malate active pool was small in the C_3_ species (85 nmol g^–1^ FW), ~70% higher in the C_3_–C_4_ species, and >10-fold higher in C_4_-like and complete C_4_ species, reaching values almost as high as in maize. The Asp active pool was relatively high in the C_3_ species (180 nmol g^–1^ FW), at least 3-fold higher in the C_3_–C_4_ and C_4_-like species, and ~3-fold lower in the C_4_ species, but still higher than in maize. The Pyr active pool was very low in the C_3_ species (4 nmol g^–1^ FW), ~80% higher in the C_3_–C_4_ species, and was dramatically higher (20- to 40-fold) in the C_4_-like and C_4_ species, where the pool resembled that in maize. In comparison with other CCM-related metabolites, the active PEP pool was small, but increased progressively from C_3_ to C_3_–C_4_, C_4_-like, and C_4_ species, with the *F. bidentis* pool resembling that in maize. The Ala active pool in the C_3_ species (80 nmol g^–1^ FW) was about half that of Asp, remained low in the C_3_–C_4_ species, and was >15-fold higher in the C_4_-like and complete C_4_ species, again like the active pool in maize.

Active pools of 2OG and Glu were low in the C_3_ species, ~2-fold higher in the C_3_–C_4_ and C_4_-like species, and Glu increased by another 2-fold in the complete C_4_ species. The estimated active pool sizes of 2OG and Glu in the C_4_*Flaveria* species resembled those reported for maize.

Among photorespiratory metabolites, the Gly active pool was low in the C_3_ species (~50 nmol g^–1^ FW), ~2-fold higher in the C_3_–C_4_ species, and low in C_4_-like and complete C_4_ species, which was about half that previously reported for maize. The Ser active pool was large in the C_3_ species (710 nmol g^–1^ FW), ~70% higher in the C_3_–C_4_ species, but considerably lower in the C_4_-like and C_4_*Flaveria* species, which resembled maize. It is noteworthy that the active pool of Ser in the C_3_–C_4_ species is 12-fold higher than that for Gly, and was also 5- to 10-fold higher than that of any other analysed metabolite in this species, except for Asp. The glycerate active pool was ~120 nmol g^–1^ FW in the C_3_ species and >3-fold higher in the C_3_–C_4_ intermediate species, which is consistent with rapid photorespiration, as well as the possibility that glycerate may contribute to shuttling of amino groups. It was very low (~60 nmol g^–1^ FW) in the C_4_-like species, as expected if photorespiration is decreased. Unexpectedly, the complete C_4_ species contained a large glycerate active pool (420 nmol g^–1^ FW). The active pool in the C_4_*Flaveria* species was ~4-fold larger than in maize, which contained an active glycerate pool similar to that in the C_3_*Flaveria* species. Finally, the active pools of 3PGA and DHAP increased progressively from C_3_ to C_3_–C_4_, C_4_-like, and C_4_*Flaveria* species, but were consistently much lower than in maize (for more analysis see the next section and the Discussion).

### Cross-species comparison of CBC intermediate profiles

Arrivault *et al*. (2019) recently presented a cross-species comparison using CBC metabolites and 2PG amounts in five C_3_ plants and four NADP-malic enzyme (NADP-ME) subtype C_4_ plants, including the two C_4_*Flaveria* species used in the current study. In PC analyses with this subset of metabolites, C_3_ species separated from C_4_ species, and there was divergence between different C_4_ species, and between different C_3_ species. These analyses pointed to changes in CBC operation between C_3_ and C_4_ plants, probably as a result of the CBC adapting to a high CO_2_ environment. We were interested to learn how *Flaveria* C_3_, C_3_–C_4_ intermediate, and C_4_-like species map onto this landscape.

**Fig. 6.**
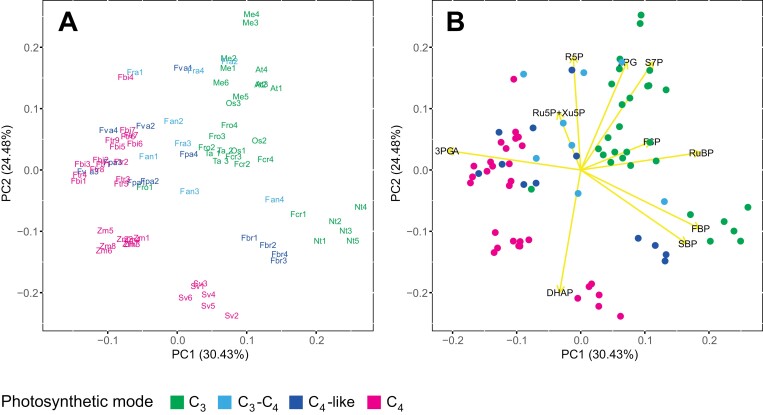
Principal component (PC) analysis of CBC metabolites and 2PG amounts, combining data from the current study of nine *Flaveria* species with data for five further C_3_ species and two further NADP-ME subtype C_4_ species. PC analysis was performed on dimensionless datasets, combining data from this study and from Arrivault *et al*. (2019). The data for CBC metabolite levels in *F. bidentis* and *F. trinervia* were already included in the analysis of Arrivault *et al*. (2019). (A) Distribution of samples along the two first two PCs, with each sample being represented by a coloured label indicating the species and biological replicate number. (B) Metabolite eigenvectors driving sample separation are shown in yellow, while individual samples appear as coloured dots. In both panels, the colour code represents the different photosynthetic modes, as indicated by the key below the figure. Species abbreviations (and photosynthesis mode) are, alphabetically: At, *Arabidopsis thaliana* (C_3_); Fan, *F. anomala* (C_3_–C_4_); Fbi, *F. bidentis* (C_4_); Fbr, *F. brownii* (C_4_-like); Fcr, *F. cronquistii* (C_3_); Fpa, *F. palmeri* (C_4_-like); Fra, *F. ramosissima* (C_3_–C_4_); Fro, *F. robusta* (C_3_); Ftr, *F. trinervia* (C_4_); Fva, *F. vaginata* (C_4_-like); Me, *Manihot esculenta* (C_3_); Nt, *Nicotiana tabacum* (C_3_); Os, *Oryza sativa* (C_3_); Sv, *Setaria viridis* (C_4_); Ta, *Triticum aestivum* (C_3_); Zm, *Zea mays* (C_4_). For metabolite abbreviations, refer to Supplementary Table S2, and for the original data see Supplementary Dataset S3.

We performed PC analysis on a combined dataset containing CBC metabolites and 2PG in the nine species from Arrivault *et al.* (2019) and the seven additional C_3_, C_3_–C_4_, and C_4_-like *Flaveria* species from the current study (Fig. 6). The analysis was performed on a dimensionless dataset (Supplementary Dataset S3). We chose this normalization method because some of the species used in Arrivault *et al.* (2019) had a high protein or chlorophyll content, leading to them being systematically displaced when PC analysis was performed with metabolites expressed on a FW basis.

PC1 and PC2 captured 30.4% and 24.5% of total variance in this large dataset (Fig. 6; see Supplementary Table S5 for the first 10 PC contributions). The distribution of the nine species in the study of Arrivault *et al.* (2019) was largely retained in this extended *Flaveria* analysis (compare Fig. 6A with fig. 4D in Arrivault *et al*., 2019). Of the seven added *Flaveria* species, the two C_3_ species lay close to other C_3_ species (especially *Triticum aestivum* and *Oryza sativa)*. The two *Flaveria* C_3_–C_4_ species and the Clade B C_4_-like species *F. brownii* fell into a diagonal between the C_3_ and complete C_4_ species (Fig. 6A). The Clade A C_4_-like species *F. palmeri* and *F. vaginata* lay close to the Clade A C_4_ species *F. bidentis* and *F. trinervia*. The Clade B C_3_–C_4_*F. anomala* showed some scatter, but most samples were closer to the Clade A C_3_–C_4_*F. ramosissima* and the two Clade A C_4_-like species than the Clade B C_4_-like species *F. brownii*. Overall, C_3_, C_3_–C_4_, C_4_-like, and complete C_4_ species were separated mainly along PC1, driven by high 3PGA and low levels of 2PG and several CBC intermediates including S7P, F6P, SBP, FBP, and, especially, RuBP (Fig. 6B). The Clade B C_4_-like *F. brownii* is somewhat of an outlier, separating from all other *Flaveria* species in PC1 and especially PC2, driven by high FBP and SBP (Fig. 6B).

## Discussion

Using a phylogenetically informed approach, we profiled >50 metabolites in nine *Flaveria* species that use different photosynthetic modes to provide new insights into the evolution of C_4_ photosynthesis in this genus from a metabolic perspective. Our study reveals substantial metabolic diversity within the genus. Importantly, hierarchical clustering (Fig. 1) and PC analysis (Fig. 2; Supplementary Fig. S2) revealed that metabolite profiles suffice to classify these nine species according to photosynthesis mode and phylogenetic relationship, providing intrinsic support for our approach.

In some cases, total metabolite content overestimates the pool that is actively involved in photosynthetic metabolism (Szecowka *et al*., 2013; Ma *et al.*, 2014; Arrivault *et al.*, 2017; Allen and Young, 2020). We therefore performed 40–60 min ^13^CO_2_ pulse labelling experiments in one basal C_3_ species and three species from Clade A, one C_3_–C_4_ intermediate, one C_4_-like, and one complete C_4_ species (Supplementary Fig. S1; McKown *et al.*, 2005; Lyu *et al.*, 2015). This allowed us to estimate the active pool sizes for a subset of the metabolites. The active pools were noticeably smaller than the total content for several metabolites, for example malate, 2OG, and Glu, as previously reported for other species (Hasunuma *et al.*, 2010; Szecowka *et al.*, 2013; Weissmann *et al.*, 2016; Arrivault *et al.*, 2017). However, total content and active pool sizes usually showed similar trends between *Flaveria* species.

**Fig. 7.**
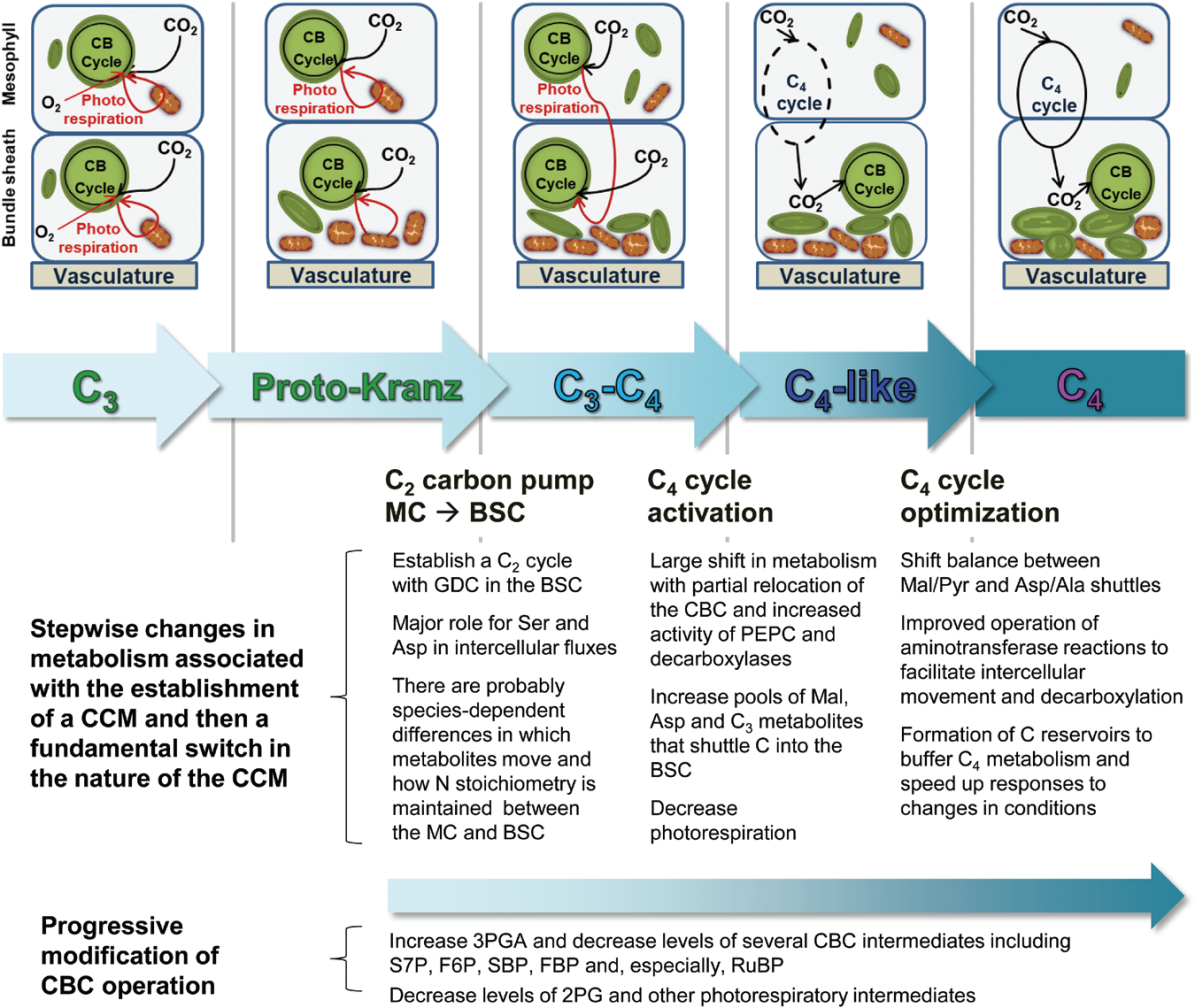
Schematic representation of stepwise changes in primary metabolism along the proposed path from C_3_ to C_4_ photosynthesis, based on current phylogeny of the genus *Flaveria.* The display builds on the work of Ku *et al.* (1983) and Monson *et al*. (1986) and resulting concepts developed in Sage *et al.* (2012, 2018). It is proposed that the photorespiration-dependent C_2_ CCM established in C_3_–C_4_ intermediate (indicated by red arrows) species may have been rather flexible, with different metabolites moving from the MC to the BSC. This would affect the extent to which glycine and possibly glycolate may move from the MC to the BSC, which in turn would affect the magnitude and direction of intercellular exchanges that are required to maintain N stoichiometry. There may also be flexibility in which organic acid/amino acid exchanges maintain N stoichiometry. At this step, PEPC activity in the MC would be needed to build up pools of C_4_ shuttle metabolites but would not be required to generate a net flux during steady-state photosynthesis. Similarly, decarboxylation would serve to reduce the levels of C_4_ metabolites or increase the levels of C_3_ metabolites, but net flux would not be needed in steady-state photosynthesis. A small draw-down of CO_2_ in the MC by PEPC would lead to increased RuBP oxygenation and strengthen the C_2_ shuttle, but a large decrease would result in a futile cycle and decreased net C fixation. This dichotomy might be broken by increased relocation of the CBC to the BSC accompanied, crucially, by increased C_4_ acid decarboxylation rates in the BSC. This shift from a C_2_ to C_4_ cycle represents a very large switch in metabolism, and would require increased PEPC activity in the MC, relocation of at least part of CBC capacity to the BSC, increased decarboxylation in the BSC, and increased levels of organic acids and other metabolites required for the C_4_ cycle. The final optimization of C_4_ photosynthesis would have included not only optimization of the expression patterns and regulation of enzymes involved in the C_4_ cycle, but also changes in the balance between different shuttles and optimization of their operation by establishing reserve pools of organic acids and amino acids. In parallel, CBC operation would have progressively adjusted to a gradual increase in the CO_2_ concentration around Rubisco. It should be noted that the scheme considers the proposed path to NADP-ME-type C_4_ photosynthesis_._ Shuttling of amino acids remains a crucial aspect of C_4_ cycles in NAD-ME- and PCK-type C_4_ species where much of the C_4_ flux is carried by Asp. Abbreviations: Ala, alanine; Asp, aspartate; BSC, bundle sheath cell; CBC, Calvin–Benson cycle; CCM, carbon-concentrating mechanism; GDC, glycine decarboxylase; MC, mesophyll cell; PEPC, phospho*enol*pyruvate carboxylase; Pyr, pyruvate; Mal, malate.

Our results provide new insights into the current model of NADP-ME-type C_4_ evolution in the genus *Flaveria*, based on early studies by Ku *et al*. (1983) and Monson *et al.* (1986) and general models of C_4_ evolution (Sage *et al*., 2012, 2018; Sage, 2016). By revealing which metabolite levels differ in species representing the stages of C_4_ evolution, clues are obtained about how nature built a C_4_ pathway (see Fig. 7). In particular, two related questions can be addressed. (i) Are the observed changes in metabolite levels those expected as new reactions are co-opted into photosynthetic C fixation? (ii) Do metabolites that are involved in predicted intercellular shuttles show an increase in content, as might be expected if intercellular movement occurs by diffusion and is driven by intercellular concentration gradients?

### From C_3_ to C_3_–C_4_ intermediacy

The two C_3_*Flaveria* in our study differ in terms of anatomy: *F. cronquistii* has classical C_3_ anatomy with abundant palisade mesophyll and inconspicuous BS cells, while *F. robusta* has a proto-Kranz anatomy, with slightly larger BS cells containing more organelles than those of *F. cronquistii* (Sage *et al.*, 2013). These C_3_ species showed differences in metabolic profiles (Figs 1, 2), including higher levels of several CBC metabolites, Gln, as well as metabolites that are unrelated to photosynthesis such as higher Lys, Thr, Tyr, Pro, and shikimate in *F. cronquistii* and higher Arg content in *F. robusta* (Fig. 3; Supplementary Fig. S3). It is unlikely that these differences are related to the evolution of C_3_–C_4_ photosynthesis and subsequent steps towards C_4_ photosynthesis.

In *Flaveria*, the transition from C_3_ to C_3_–C_4_ photosynthesis has been proposed to involve a shift of GDC activity from the mesophyll to the BS cells, leading to the establishment of a photorespiratory CO_2_ pump in which Gly moves from the mesophyll to the BS, where it is decarboxylated, generating an elevated CO_2_ concentration, and Ser moves back to the mesophyll (see the Introduction; Fig. 7). The NH_3_ released by GDC is assimilated by the GOGAT pathway in the BS, and an intercellular shuttle is required to return an amino acid to the mesophyll and recycle an organic acid to the BS (Rawsthorne *et al.*, 1988*b*; Rawsthorne and Hylton, 1991; Gowik *et al.*, 2011; Mallmann *et al.*, 2014).

We had expected to find an increase in Gly and Ser levels in C_3_–C_4_ intermediate *Flaveria* species relative to C_3_ species that would support a photorespiratory CO_2_ pump. However, total Gly content (Fig. 3C) and the active Gly pool (95 nmol g^–1^ FW; Fig. 5) were low in C_3_ species and were not consistently higher in C_3_–C_4_ species. Gly was previously reported to be low in the Clade B C_3_–C_4_ intermediate species *F. floridiana*, whereas it was high in the C_3_–C_4_ intermediate species, *Moricandia arvensis* (Leegood and von Caemmerer, 1994). Ser was present at >10-fold higher levels than Gly in the C_3_ species (Fig. 3C), and the absolute (both) and active (*F. ramosissima*) pool of Ser was noticeably even higher in the C_3_–C_4_ intermediates (Figs 3C, 5). These high Ser levels are consistent with a C_2_ cycle in which Ser diffuses from the BS to the mesophyll; but questions remain with regard to movement of Gly from the mesophyll to the BS (see below for further discussion). That said, Ser was not noticeably higher than Gly in an earlier study of the C_3_–C_4_ intermediate species *F. floridiana* and *M. arvensis* (Leegood and von Caemmerer, 1994).

There were no consistent or marked increases between the C_3_ and C_3_–C_4_ species in the levels of organic acids and amino acids that might be involved in a shuttle that maintains N stoichiometry between the BS and mesophyll. However, *F. anomala* and *F. ramosissima* are in different clades of the *Flaveria* phylogeny (Supplementary Fig. S1; McKown *et al.*, 2005; Lyu *et al.*, 2015), indicating that they represent independent origins of C_3_–C_4_ photosynthesis. Also, although both species were classified as type II intermediates by Mallmann *et al.* (2014), they may represent slightly different stages in the establishment of C_3_–C_4_ photosynthesis. *Flaveria anomola* has sometimes been considered an early C_3_–C_4_ intermediate (Moore *et al.*, 1987), whereas *F. ramosissima* is an advanced intermediate with relatively high PEPC and Asp aminotransferase (AspAT) activities (Ku *et al.*, 1983, 1991; Bauwe, 1984), and has been reported to assimilate up to 50% of its C through PEPC (Moore *et al.*, 1987; Chastain and Chollet, 1989). We therefore inspected the metabolite profiles in *F. ramosissima* and *F. anomala* separately.

The active pool of Ser in *F. ramosissima* (~1200 nmol g^–1^ FW; Fig. 5) might be taken as a benchmark for the pool size required to support formation of a concentration gradient large enough to drive intercellular movement at a rate that balances N between the BS and mesophyll. Among organic acids and amino acids that might be involved in intercellular shuttles in *F. ramosissima*, the largest active pool was found for Asp (Fig. 5). Whilst the total pool of Asp did not differ between C_3_ and C_3_–C_4_ species (Fig. 3A), due to much higher enrichment, the active pool was ~4-fold larger in *F. ramosissima* than in *F. robusta* (Fig. 5), reaching a level (~750 nmol g^–1^ FW) that was about two-thirds of the *F. ramosissima* Ser active pool. The active pools of malate and Ala were several times smaller, and that of Pyr was very small compared with the active Ser pool in *F. ramosissima* (~8, 10, and 200 times smaller, respectively; Fig. 5). The total malate content in *F. ramosissima* was similar to or slightly lower than in C_3_ species (Fig. 3A), and the active pool (~150 nmol g^–1^ FW; Fig. 5) showed at the most only a slight increase compared with the C_3_ species *F. robusta*, and was ~10-fold smaller than the malate active pool in C_4_-like and complete C_4_*Flaveria* species (Fig. 5). The overall (Fig. 3) and active (Fig. 5) pools of Ala did not differ markedly between C_3_ species and *F. ramosissima*, and the active pool (~120 nmol g^–1^ FW; Fig 5) was >10 times smaller than in C_4_-like and complete C_4_ species (Figs 3A, 5). The total (Fig. 3) and active (Fig. 5) pools of Pyr in *F. ramosissima* resembled those in C_3_ species, and were ~3- and >10-fold smaller than the corresponding Pyr pools in C_4_-like species, and even smaller compared with those in complete C_4_ species. The 2OG content and active pool were very low in C_3_ species and, although the active pool was twice as large in *F. ramosissima*, it was also low in C_4_-like and complete C_4_ species (130 nmol g^–1^ FW; Figs 3, 5). Taken together, our results are consistent with the idea that high PEPC and AspAT activities in *F. ramosissima* (Ku *et al.*, 1983, 1991; Bauwe, 1984) lead to formation of Asp, which acts as a major N equilibrator between the BS and mesophyll cells. They fit with a submodel of the C_2_ cycle that has a constraint on intercellular movement of Glu, 2OG, Ala, and Pyr (Mallmann *et al.*, 2014). However, our analyses do not identify a clear single candidate for the C skeleton that moves from the mesophyll to the BS. Further, they do not provide strong evidence for rapid malate/Ala exchange, although slow exchange might be supported without increasing the Ala pool much above that in C_3_ plants.

Relative to the Clade A C_3_–C_4_ intermediate *F. ramosissima*, the Clade B C_3_–C_4_ intermediate *F. anomala* had much higher overall levels of Gln and Asn (~600 nmol g^–1^ FW and 900 nmol g^–1^ FW, respectively) and relatively high Asp and Glu, but a trend to even lower malate, Pyr, 2OG, and Ala amounts (Fig. 3A). An earlier study of another Clade B C_3_–C_4_ intermediate, *F. floridiana*, revealed slightly higher Asp, as well as Pyr, relative to the C_3_*F. pringlei* (Leegood and von Caemmerer, 1994). The most plausible candidates for intercellular shuttles to transfer amino groups in C_3_–C_4_ intermediate species are a Gln/Glu or an Asn/Asp exchange.

Altogether, our data for both studied C_3_–C_4_ species are consistent with the operation of a C_2_ cycle in which Ser diffuses from the BS to the mesophyll (Fig. 7). However, Gly levels are low and there is no consistent increase of Gly in the C_3_–C_4_ species compared with the C_3_ species. This raises the question of whether earlier metabolites in the photorespiratory pathway, such as glycolate, also move from the mesophyll to the BS cells, which in turn would decrease the need to recycle amino groups to the mesophyll. An earlier study of *M. arvensis* and *F. floridana* (Leegood and von Caemmerer, 1994) points to C_3_–C_4_ species in separate lineages differing in which C_2_ metabolite moves from the mesophyll to the BS cells. Our data also point to different sets of metabolites being involved in maintaining N balance in the two C_3_–C_4_ species studied here, with Clade A *F. ramosissima* using mainly Asp and Clade B *F. anomala* using Gln or Asn to return amino groups to the mesophyll. Collectively, our data and those of Leegood and von Caemmerer (1994) indicate that multiple metabolites may have been co-opted as carriers during the C_3_ to C_3_–C_4_ intermediate transition (Fig. 7), in which case the overall level of single metabolite pools would not need to increase markedly. This fits with the general similarity of the metabolite profiles of C_3_ and C_3_–C_4_ species, as seen in our clustering and PC analyses.

### From C_3_–C_4_ intermediacy to C_4_-like

Advanced C_3_–C_4_ species such as *F. ramosissima* possess elements of a C_4_-like CCM, with increased PEPC activity relative to C_3_ and Type I C_3_–C_4_ intermediate species. The transition from a C_3_–C_4_ pathway to a C_4_-like state is thought to have involved a gradual shift of Rubisco from the mesophyll to BS cells and increased enzymatic activity in the later part of the C_4_ cycle, especially pyruvate,orthophosphate dikinase (PPDK) (Ku *et al.*, 1983) to boost regeneration of PEP. It would also require increased decarboxylation of C_4_ metabolites in the BS.

Co-opting multiple metabolites to the intercellular shuttles in C_3_–C_4_ photosynthesis (see previous section) might have provided important metabolic flexibility that increased the chance of establishing a primitive C_4_ cycle. For example, movement of glycolate in parallel with Gly would decrease the need to shuttle amino groups back from the BS cells to the mesophyll; indeed, if more glycolate were to move than glycine, amino groups would need to be shuttled from the BS to the mesophyll. This would create a set of alternative scenarios where a primitive C_4_ cycle could operate simultaneously with a C_2_ cycle. Hypothetical options would include not only an Asp/Pyr exchange that transfers amino groups to the BS, but also amino group-neutral Asp/Ala or malate/Pyr exchanges, or even a malate/Ala cycle with net transfer of amino groups from the BS to the mesophyll.

Our metabolite data reveal a substantial change in the primary metabolome of C_4_-like species compared with C_3_–C_4_ intermediates. This is clearly displayed in the clustering and PC analyses (Figs 1, 2). C_4_-like species differ from C_3_–C_4_ intermediate species by having higher contents of C_4_-related metabolites, such as malate, Pyr, and Ala, and generally lower contents of photorespiratory intermediates, such as Ser and glycerate (Fig. 3). Our labelling analyses on the C_4_-like species *F. palmeri* suggest that this evolutionary step involved a large increase in the active pools of malate, Pyr, and Ala (Fig. 5), providing strong support for the operation of an expanded NADP-ME-type C_4_ cycle with integration of aminotransferases (Fig. 7; Furbank, 2011; Schlüter *et al.*, 2018). Our analyses also point to increased exchange of C between the CBC and the C1–C3 positions of the carrier metabolites, presumably as a result of increased PEPC activity. Whilst Asp levels are slightly lower in *F. palmeri* than in *F. ramosissima* (Figs 3, 5), they may be sufficient to support some intercellular movement of Asp. Engelmann *et al.* (2003) suggested that a switch from Asp to malate as a carrier happened soon after the transition from C_3_–C_4_ to C_4_-like photosynthesis; malate has the advantage that its decarboxylation by NADP-ME provides the BS cells with reducing equivalents, which can power reactions in the CBC.

There were noticeably higher levels of Asn in C_4_-like species than in C_3_ and C_3_–C_4_ intermediates, and these high levels are in part seen in complete C_4_ species (Supplementary Fig. S3). Asn represents a potential N-rich compound for shuttling (Mallmann *et al.*, 2014), possibly in combination with Asp or malate. ^13^CO_2_ labelling data for Asn would be required to provide direct evidence for its involvement, including assessing if the rate of labelling resembles that of Asp and malate, and determining the size of the active pool.

The overall levels of photorespiratory metabolites such as Gly, Ser, and glycerate (Fig. 3C) tended to be lower in C_4_-like species than in C_3_ and C_3_–C_4_ species. The active pools of Gly and Ser were also consistently smaller in *F. palmeri* than in *F. ramosissima* (Fig. 5), indicating that C_4_-like species do not employ a C_2_ cycle and instead benefit from lower rates of photorespiration (Ku *et al.*, 1991).

In summary, our results point to a major reorganization of metabolism between C_3_–C_4_ and C_4_-like photosynthesis, including large increases in the pools of malate, Pyr, and Ala, retention of a large pool of Asp, and an increase in the pool of Asn. This is consistent with the establishment of a mixed C_4_-like CCM involving both organic acids and amino acids (Fig. 7). The decrease in levels of photorespiratory metabolites is consistent with a substantial decrease in photorespiration.

### From C_4_-like to complete C_4_

The last step in the evolution of C_4_ photosynthesis is proposed to have included optimization of the compartmentation and the properties of key CCM-related enzymes such as Rubisco and PEPC (Kubien *et al.*, 2008; Gowik and Westhoff, 2011; Kapralov *et al.*, 2011). Our metabolite analyses capture further changes that probably occurred in parallel. One appears to be a refinement of the NADP-ME-type C_4_ cycle toward one that is less reliant on aminotransferase reactions (Fig. 7). Compared with C_4_-like species, complete C_4_ species show a trend to higher malate and Pyr content, a 2- to 3-fold lower amount of Asp, and a trend to lower levels of Ala (Fig. 3A). Labelling studies revealed that, compared with C_4_-like *F. palmeri*, the complete C_4_ species *F. bidentis* contained a similar or marginally higher active malate pool, an almost 2-fold higher active Pyr pool, and smaller active pools of Asp and Ala (Fig. 5).

Another striking shift between C_4_-like species and those with a complete C_4_ syndrome was an increase in the levels of several TCA cycle intermediates (citrate, isocitrate, aconitate, and 2OG) (Figs 1, 2, 3A; Supplementary Figs S2, S3). Although the total Glu pool did not increase, more ^13^C moved into Glu in the C_4_ plant *F. bidentis* than in C_4_-like *F. palmeri* (Fig. 5; Supplementary Fig. S4), indicating increased flux through the TCA cycle to this amino acid. It is notable that levels of 2OG and Glu in *F. bidentis* resembled those in the model NADP-ME-type C_4_ species maize (Fig. 5). One possible role for 2OG and Glu in C_4_ photosynthesis might be to support flux through AspAT or Ala aminotransferase in the mesophyll or the BS cells. This might have contributed to the decline in Ala and especially Asp in the transition from C_4_-like to C_4_ photosynthesis in *Flaveria* (Fig. 7). It might be noted that for participation of Glu and 2OG in aminotransferase reactions, the overall rather than the active pool (as defined by incorporation of ^13^C) may be more relevant. At least in steady-state conditions, this function can be supported by existing pools, as the C skeleton of Glu and 2OG is recycled during the complete amino transfer cycle and does not need to be synthesized *de novo* from newly fixed C.

A further interesting change in the transition from C_4_-like to C_4_ in *Flaveria* concerns glycerate, whose level is higher in complete C_4_ species than in C_4_-like species (Fig. 3C). Glycerate presumably has different roles in the C_4_ and C_3_ pathways, as photorespiration is greatly reduced in C_4_ plants. One possibility is that glycerate acts as a C reserve that can be used to replenish the levels of CBC- and CCM-related metabolites (Usuda and Edwards, 1980; Weber and von Caemmerer, 2010), for example in transitions between different C availability states (Levey *et al.*, 2019).

Overall, our results indicate that, in addition to changes in the location and properties of enzymes, optimization of C_4_ photosynthesis in *Flaveria* also involved (i) a shift towards increased use of a malate/Pyr compared with an Asp/Ala exchange, implying that NADP-ME makes an increasing contribution to decarboxylation compared with other decarboxylation pathways; (ii) an increase in the overall levels of 2OG that might potentially aid aminotransferase reactions in one or both cell types; and (iii) an increase in the levels of TCA cycle acids and glycerate that might serve as C reservoirs to buffer against fluctuations in the environment.

### Clade-dependent metabolite differences and considerations

The absence of complete C_4_ species in *Flaveria* Clade B raises questions about the evolution of C_4_ photosynthesis in the genus. Does it reflect a difference between the two clades in the timing of the evolutionary process where Clade B species are younger and none has yet had time to complete the passage to C_4_ photosynthesis, or are Clade B species not found in habitats that favour C_4_ photosynthesis, or was C_3_-C_4_ or C_4_-like photosynthesis established in Clade B using a combination of biochemical pathways that was unfavourable for evolution of a complete C_4_ syndrome, such as the lack of genetic enablers (Christin and Osborne, 2014)? The finding that the Clade B C_3_–C_4_*F. anomola* and C_4_-like *F. brownii* formed a distinct cluster from all Clade A species (Fig. 1) is consistent with them having a different balance of biochemical pathways. Curiously, in the PC analysis of Fig. 2, the Clade A C_3_–C_4_ intermediate *F. ramosissima* grouped quite close to the basal C_3_ species, and was clearly separated from the Clade B C_3_–C_4_*F. anomola* that grouped closer to the C_4_-like species, including those in Clade A. This indicates that if there are clade-dependent differences in metabolism that hinder the move towards full C_4_ photosynthesis, they may be rather specific.

As already mentioned, the Clade B C_3_–C_4_ intermediate *F. anomala* may use Gln/Glu or Asn/Asp shuttles to return an amino acid to the mesophyll and recycle an organic acid to the BS. This might be less conducive to evolution of a PEPC-based CCM than a shuttle based on Asp and one or a mix of organic acids. Separation of the Clade B C_4_-like species *F. brownii* from the Clade A C_4_-like species was also driven by high amino acid levels, including Ala and Asn (Fig. 2). However, Ala, Asn, and in one case (C_4_-like *F. vaginata*) Gln levels were higher in C_4_-like than in C_3_–C_4_ species in Clade A, indicating that high levels of these amino acids do not *per se* preclude evolution of full C_4_ photosynthesis. Other clade-dependent differences included a trend to higher RuBP, FBP, and SBP in Clade B species. It is not obvious how this would affect the propensity to evolve a complete C_4_ pathway.

While our analyses reveal clade-dependent differences in metabolism, too few species were studied to conclusively identify specific features that might hinder evolution of complete C_4_ photosynthesis in Clade B. It is also noteworthy that *F. brownii* dampens its C_4_ cycle at low irradiance (Cheng *et al.*, 1989). This observation is consistent with the idea that the gains from C_4_-like photosynthesis in this species are not large, especially in low irradiance when the cost of operating its CCM may be high compared with available light energy.

### Changes in CBC operation along the evolutionary path from C_3_ to C_4_ photosynthesis

It is well established that CBC operation differs between C_3_ and C_4_ species (see the Introduction). Recently, profiling of CBC metabolites revealed widespread differences in levels between C_4_ and C_3_ species, between different C_4_ species, and between different C_3_ species (Arrivault *et al.*, 2019; Borghi *et al.*, 2019). This indicates that in addition to adjustment of the CBC during the evolution of C_4_ photosynthesis, there is also interspecies diversity in how the CBC operates in different C_3_ species and in different C_4_ species. This diversity may be relevant to current attempts to engineer C_4_ photosynthesis into C_3_ crops, and to exploit photosynthetic diversity for improving crops (Lawson *et al.*, 2012; Driever *et al.*, 2014; Simkin *et al.*, 2019).

To investigate how CBC operation was modified during the evolutionary progression from C_3_ to C_4_ photosynthesis, we used PC analysis to compare CBC metabolite profiles in the nine *Flaveria* species and in several C_3_ and C_4_ species from outside the genus (Fig. 6). These analyses showed that it is possible to separate plants with differing photosynthetic strategies on the basis of their CBC metabolite profile. The C_3_*Flaveria* species have a CBC profile close to that of the other C_3_ species examined, especially rice and wheat. Intermediate *Flaveria* species lie on the path between C_3_ and C_4_ species, with the C_3_–C_4_ intermediate species being closer to C_3_ species and C_4_-like species closer to complete C_4_ species. Further, diversity in CBC function may exist between *Flaveria* clades, as the metabolic profiles of the Clade B C_4_-like *F. brownii* and C_3_–C_4_*F. anomala* differ from those of Clade A C_4_-like and C_3_–C_4_ species. More generally, these results indicate that CBC operation had already started to adapt to enhanced internal CO_2_ status in the C_3_–C_4_*Flaveria* species and that this adaptation progressed furthest in C_4_-like and C_4_ species in Clade A.

The evolutionary transition from C_3_ to C_4_ photosynthesis was mainly captured in PC1, and was driven by increasing 3PGA and decreasing levels of 2PG and several CBC intermediates including S7P, F6P, SBP, FBP, and, especially, RuBP. RuBP is highest in C_3_ and C_3_–C_4_ species, and decreases through C_4_-like to C_4_ species (Fig. 3B). Evolution of C_4_ photosynthesis is accompanied by a decrease in Rubisco abundance, made possible by relaxed selection for catalytic fidelity for CO_2_ and an associated increase in *k*_cat_ (Sage, 2002; Ghannoum *et al.*, 2005; Kapralov *et al.*, 2011). As most RuBP is bound in the active site of Rubisco (Salvucci, 1989), the lower RuBP may in part reflect the lower Rubisco abundance. Lower 2PG presumably reflects the decrease in photorespiration.

The trend to higher 3PGA in C_4_-like and C_4_ species may be linked to operation of a 3PGA/DHAP shuttle between mesophyll and BS cells (Leegood, 1985; Stitt and Heldt, 1985; Arrivault *et al.*, 2017). High levels of both 3PGA and DHAP are found in *Setaria viridis* and maize (see Fig. 5). This shuttle allows BS chloroplasts to outsource part of the reductive phase of the CBC to the mesophyll chloroplasts where ATP and NADPH are readily available, and is required in NADP-ME-type C_4_ species such as maize that have dimorphic chloroplasts and little or no PSII activity in the BS cells (Woo *et al.*, 1970; Andersen *et al.*, 1972; Munekage, 2016). It is still unproven if *S. viridis* makes use of this shuttle and has the same chloroplastic properties as maize, but our metabolite data suggest this is the case. The C_4_*Flaveria* species have PSII activity in their BS chloroplasts to a varying extent, depending on conditions (Laetsch and Price, 1969; Höfer *et al.*, 1992; Meister *et al.*, 1996; Nakamura *et al.*, 2013). There is only a weak trend to higher 3PGA in *Flaveria* C_4_-like and C_4_ species (Fig. 6; see also Fig. 3B). This might reflect partial operation of an intercellular 3PGA/DHAP shuttle (Leegood and von Caemmerer, 1994).

### General conclusions

Our comparative analysis of metabolite levels in nine *Flaveria* species representing steps along the evolutionary path from C_3_ to complete C_4_ photosynthesis supports and extends current ideas about how C_4_ photosynthesis evolved through several semi-stable stages (Fig. 7). At the level of metabolism, the first step was preferential location of GDC activity to the BS cells and the associated appearance of intercellular shuttles of photorespiratory metabolites. High levels of Ser in C_3_–C_4_*Flaveria* species support the idea that this metabolite moves from the BS to the mesophyll cells following GDC activity. However, whilst Asp levels were high, we did not find substantial increases in the levels of Gly or of other metabolites that are required to maintain N stoichiometry between the mesophyll and BS, indicating that intercellular fluxes may be partitioned between two or more metabolites. The transition from C_3_–C_4_ to a C_4_-like state was associated with a major change in metabolism, including clear increases of malate and three-C metabolites that are required to shuttle CO_2_ into the BS cells. The transition from C_4_-like to a complete C_4_ syndrome was associated with changes in metabolites indicative of a shift in the balance between malate/Pyr and Asp/Ala shuttles, improved operation of aminotransferase reactions, and the formation of C reservoirs to buffer C_4_ metabolism against fluctuations in the environment. In addition, focused analysis of CBC metabolites revealed progressive adaptation of the CBC to an increasingly effective CCM.

## Supplementary data

The following supplementary data are available at *JXB* online.

Fig. S1. Phylogenetic tree of 16 *Flaveria* species.

Fig. S2. Principal component (PC) analyses of metabolite profiles in nine *Flaveria* species: PC1 in combination with PC3 or PC4.

Fig. S3. Absolute amounts of additional metabolites in nine *Flaveria* species.

Fig. S4. ^13^C enrichment in key metabolites in four Clade A *Flaveria* species with different modes of photosynthesis.

Table S1. Germination, growth, and harvest conditions.

Table S2. List of measured metabolites, their abbreviations, and analytical methods used.

Table S3. Summary of principal components for analyses on the entire *Flaveria* dataset.

Table S4. Use of metabolite amounts and ^13^C enrichments to estimate active and inactive pools in four *Flaveria* species and maize.

Table S5. Summary of first 10 PCs for PC analyses on the dimensionless multispecies dataset for CBC metabolites and 2PG.

Dataset S1. Metabolite amounts in nine *Flaveria* species.

Dataset S2. ^13^C enrichment (%) in a selection of metabolites in four *Flaveria* species.

Dataset S3. Metabolite amounts of CBC intermediates plus 2PG in nine *Flaveria* species combined with data for another five C_3_ species and two C_4_ species from Arrivault *et al.* (2019).

erab540_suppl_supplementary_dataset_S1-S3

erab540_suppl_supplementary_figures_S1-S4_tables_S2-S5

erab540_suppl_supplementary_table_S1

## Data Availability

All data supporting the findings of this study are available within the paper and within its supplementary data published online.

## Abbreviations

BS: bundle sheath
CA: carbonic anhydrase
CBC: Calvin–Benson cycle
CCM: CO_2_-concentrating mechanism
GC-TOF-MS: GC coupled with time-of-flight MS
GDC: glycine decarboxylase
GOGAT: glutamine–oxoglutarate aminotransferase
NADP-ME: NADP-dependent malic enzyme
PC: principal component
PCK: phosphoenolpyruvate carboxykinase
PEPC: phosphoenolpyruvate carboxylase
RH: relative humidity
PPM®: Plant Preservation Medium
TCA: tricarboxylic acid

## References

[CIT0001] Allen DK , Young JD. 2020. Tracing metabolic flux through time and space with isotope labeling experiments. Current Opinion in Biotechnology 64, 92–100. 31864070 10.1016/j.copbio.2019.11.003 PMC7302994

[CIT0002] Andersen KS , Bain JM, Bishop DG, Smillie RM. 1972. Photosystem II activity in agranal bundle sheath chloroplasts from *Zea mays*. Plant Physiology 49, 461–466. 16657984 10.1104/pp.49.4.461 PMC365988

[CIT0003] Arrivault S , Alexandre Moraes T, Obata T, et al. 2019. Metabolite profiles reveal interspecific variation in operation of the Calvin–Benson cycle in both C_4_ and C_3_ plants. Journal of Experimental Botany 70, 1843–1858. 30773587 10.1093/jxb/erz051 PMC6436152

[CIT0004] Arrivault S , Guenther M, Fry SC, Fuenfgeld MM, Veyel D, Mettler-Altmann T, Stitt M, Lunn JE. 2015. Synthesis and use of stable-isotope-labeled internal standards for quantification of phosphorylated metabolites by LC-MS/MS. Analytical Chemistry 87, 6896–6904. 26010726 10.1021/acs.analchem.5b01387

[CIT0005] Arrivault S , Guenther M, Ivakov A, Feil R, Vosloh D, van Dongen JT, Sulpice R, Stitt M. 2009. Use of reverse-phase liquid chromatography, linked to tandem mass spectrometry, to profile the Calvin cycle and other metabolic intermediates in Arabidopsis rosettes at different carbon dioxide concentrations. The Plant Journal 59, 826–839. 19453453 10.1111/j.1365-313X.2009.03902.x

[CIT0006] Arrivault S , Obata T, Szecówka M, Mengin V, Guenther M, Hoehne M, Fernie AR, Stitt M. 2017. Metabolite pools and carbon flow during C_4_ photosynthesis in maize: ^13^CO_2_ labeling kinetics and cell type fractionation. Journal of Experimental Botany 68, 283–298. 27834209 10.1093/jxb/erw414 PMC5853532

[CIT0007] Aubry S , Brown NJ, Hibberd JM. 2011. The role of proteins in C_3_ plants prior to their recruitment into the C_4_ pathway. Journal of Experimental Botany 62, 3049–3059. 21321052 10.1093/jxb/err012

[CIT0008] Bauwe H. 1984. Photosynthetic enzyme activities and immunofluorescence studies on the localization of ribulose-1,5-bisphosphate carboxylase/oxygenase in leaves of C_3_, C_4_, and C_3_–C_4_ intermediate species of *Flaveria* (Asteraceae). Biochemie und Physiologie der Pflanzen 179, 253–268.

[CIT0009] Bauwe H , Hagemann M, Fernie AR. 2010. Photorespiration: players, partners and origin. Trends in Plant Science 15, 330–336. 20403720 10.1016/j.tplants.2010.03.006

[CIT0010] Bauwe H , Kolukisaoglu U. 2003. Genetic manipulation of glycine decarboxylation. Journal of Experimental Botany 54, 1523–1535. 12730263 10.1093/jxb/erg171

[CIT0011] Bohley K , Schröder T, Kesselmeier J, Ludwig M, Kadereit G. 2019. C_4_-like photosynthesis and the effects of leaf senescence on C_4_-like physiology in *Sesuvium sesuvioides* (Aizoaceae). Journal of Experimental Botany 70, 1553–1565. 30689935 10.1093/jxb/erz011 PMC6411375

[CIT0012] Borghi GL , Moraes TA, Günther M, Feil R, Mengin V, Lunn JE, Stitt M, Arrivault S. 2019. Relationship between irradiance and levels of Calvin–Benson cycle and other intermediates in the model eudicot Arabidopsis and the model monocot rice. Journal of Experimental Botany 70, 5809–5825. 31353406 10.1093/jxb/erz346 PMC6812724

[CIT0013] Brown RH. 1978. A difference in N use efficiency in C_3_ and C_4_ plants and its implications in adaptation and evolution. Crop Science 18, 93–98.

[CIT0014] Busch FA , Sage TL, Cousins AB, Sage RF. 2013. C_3_ plants enhance rates of photosynthesis by reassimilating photorespired and respired CO_2_. Plant, Cell & Environment 36, 200–212. 10.1111/j.1365-3040.2012.02567.x 22734462

[CIT0015] Carillo P , Mastrolonardo G, Nacca F, Fuggi A. 2005. Nitrate reductase in durum wheat seedlings as affected by nitrate nutrition and salinity. Functional Plant Biology 32, 209–219. 32689125 10.1071/FP04184

[CIT0016] Chastain CJ , Chollet R. 1989. Interspecific variation in assimilation of ^14^CO_2_ into C_4_ acids by leaves of C_3_, C_4_ and C_3_–C_4_ intermediate *Flaveria* species near the CO_2_ compensation concentration. Planta 179, 81–88. 24201425 10.1007/BF00395774

[CIT0017] Cheng SH , Moore BD, Edwards GE, Ku MS. 1988. Photosynthesis in *Flaveria brownii*, a C_4_-like species: leaf anatomy, characteristics of CO_2_ exchange, compartmentation of photosynthetic enzymes, and metabolism of CO_2_. Plant Physiology 87, 867–873. 16666239 10.1104/pp.87.4.867 PMC1054860

[CIT0018] Cheng SH , Moore BD, Wu J, Edwards GE, Ku MS. 1989. Photosynthetic plasticity in *Flaveria brownii*: growth irradiance and the expression of C_4_ photosynthesis. Plant Physiology 89, 1129–1135. 16666675 10.1104/pp.89.4.1129 PMC1055986

[CIT0019] Christin PA , Besnard G, Samaritani E, Duvall MR, Hodkinson TR, Savolainen V, Salamin N. 2008. Oligocene CO_2_ decline promoted C_4_ photosynthesis in grasses. Current Biology 18, 37–43. 18160293 10.1016/j.cub.2007.11.058

[CIT0020] Christin PA , Osborne CP. 2014. The evolutionary ecology of C_4_ plants. New Phytologist 204, 765–781. 10.1111/nph.13033 25263843

[CIT0021] Danila FR , Quick WP, White RG, Furbank RT, von Caemmerer S. 2016. The metabolite pathway between bundle sheath and mesophyll: quantification of plasmodesmata in leaves of C_3_ and C_4_ monocots. The Plant Cell 28, 1461–1471. 27288224 10.1105/tpc.16.00155 PMC4944413

[CIT0022] Danila FR , Quick WP, White RG, Kelly S, von Caemmerer S, Furbank RT. 2018. Multiple mechanisms for enhanced plasmodesmata density in disparate subtypes of C_4_ grasses. Journal of Experimental Botany 69, 1135–1145. 29300922 10.1093/jxb/erx456 PMC6018992

[CIT0023] Driever SM , Lawson T, Andralojc PJ, Raines CA, Parry MA. 2014. Natural variation in photosynthetic capacity, growth, and yield in 64 field-grown wheat genotypes. Journal of Experimental Botany 65, 4959–4973. 24963002 10.1093/jxb/eru253 PMC4144772

[CIT0024] Engelmann S , Bläsing OE, Gowik U, Svensson P, Westhoff P. 2003. Molecular evolution of C_4_ phosphoenolpyruvate carboxylase in the genus Flaveria—a gradual increase from C_3_ to C_4_ characteristics. Planta 217, 717–725. 12811556 10.1007/s00425-003-1045-0

[CIT0025] Ermakova M , Arrivault S, Giuliani R, et al. 2020. Installation of C_4_ photosynthetic pathway enzymes in rice using a single construct. Plant Biotechnology Journal 19, 575–588. 33016576 10.1111/pbi.13487 PMC7955876

[CIT0026] Ermakova M , Danila FR, Furbank RT, von Caemmerer S. 2019. On the road to C_4_ rice: advances and perspectives. The Plant Journal 101, 940–950. 31596523 10.1111/tpj.14562 PMC7065233

[CIT0027] Furbank RT. 2011. Evolution of the C_4_ photosynthetic mechanism: are there really three C_4_ acid decarboxylation types? Journal of Experimental Botany 62, 3103–3108. 21511901 10.1093/jxb/err080

[CIT0028] Ghannoum O. 2009. C_4_ photosynthesis and water stress. Annals of Botany 103, 635–644. 18552367 10.1093/aob/mcn093 PMC2707343

[CIT0029] Ghannoum O , Evans JR, Chow WS, Andrews TJ, Conroy JP, von Caemmerer S. 2005. Faster Rubisco is the key to superior nitrogen-use efficiency in NADP-malic enzyme relative to NAD-malic enzyme C_4_ grasses. Plant Physiology 137, 638–650. 15665246 10.1104/pp.104.054759 PMC1065364

[CIT0030] Ghannoum O , Evans JR, von Caemmerer S. 2011. Nitrogen and water use efficiency of C_4_ plants. In: Raghavendra AS, Sage RF, eds. C_4_ photosynthesis and related CO_2_ concentrating mechanisms. Dordrecht: Springer, 129–146.

[CIT0031] Gowik U , Bräutigam A, Weber KL, Weber AP, Westhoff P. 2011. Evolution of C_4_ photosynthesis in the genus *Flaveria*: how many and which genes does it take to make C_4_? The Plant Cell 23, 2087–2105. 21705644 10.1105/tpc.111.086264 PMC3160039

[CIT0032] Gowik U , Westhoff P. 2011b. C_4_ phosphoenolpyruvate carboxylase. In: Raghavendra AS, Sage RF, eds. C_4_ photosynthesis and related CO_2_ concentrating mechanisms. Dordrecht: Springer, 257–275.

[CIT0033] Hasunuma T , Harada K, Miyazawa S, Kondo A, Fukusaki E, Miyake C. 2010. Metabolic turnover analysis by a combination of in vivo ^13^C-labelling from ^13^CO_2_ and metabolic profiling with CE-MS/MS reveals rate-limiting steps of the C_3_ photosynthetic pathway in *Nicotiana tabacum* leaves. Journal of Experimental Botany 61, 1041–1051. 20026474 10.1093/jxb/erp374 PMC2826653

[CIT0034] Häusler RE , Hirsch HJ, Kreuzaler F, Peterhänsel C. 2002. Overexpression of C_4_-cycle enzymes in transgenic C_3_ plants: a biotechnological approach to improve C_3_-photosynthesis. Journal of Experimental Botany 53, 591–607. 11886879 10.1093/jexbot/53.369.591

[CIT0035] Heckmann D. 2016. C_4_ photosynthesis evolution: the conditional Mt. Fuji. Current Opinion in Plant Biology 31, 149–154. 27153468 10.1016/j.pbi.2016.04.008

[CIT0036] Heckmann D , Schulze S, Denton A, Gowik U, Westhoff P, Weber AP, Lercher MJ. 2013. Predicting C_4_ photosynthesis evolution: modular, individually adaptive steps on a Mount Fuji fitness landscape. Cell 153, 1579–1588. 23791184 10.1016/j.cell.2013.04.058

[CIT0037] Höfer MU , Santore UJ, Westhoff P. 1992. Differential accumulation of the 10-, 16- and 23-kDa peripheral components of the water-splitting complex of photosystem II in mesophyll and bundle-sheath chloroplasts of the dicotyledonous C4 plant *Flaveria trinervia* (Spreng.) C. Mohr. Planta 186, 304–312. 24186670 10.1007/BF00196260

[CIT0038] Huege J , Goetze J, Dethloff F, Junker B, Kopka J. 2014. Quantification of stable isotope label in metabolites via mass spectrometry. Methods in Molecular Biology 1056, 213–223. 24306876 10.1007/978-1-62703-592-7_20

[CIT0039] Hylton CM , Rawsthorne S, Smith AM, Jones DA, Woolhouse HW. 1988. Glycine decarboxylase is confined to the bundle-sheath cells of leaves of C_3_–C_4_ intermediate species. Planta 175, 452–459. 24221925 10.1007/BF00393064

[CIT0040] Ishihara H , Obata T, Sulpice R, Fernie AR, Stitt M. 2015. Quantifying protein synthesis and degradation in Arabidopsis by dynamic ^13^CO_2_ labeling and analysis of enrichment in individual amino acids in their free pools and in protein. Plant Physiology 168, 74–93. 25810096 10.1104/pp.15.00209 PMC4424029

[CIT0041] Kapralov MV , Kubien DS, Andersson I, Filatov DA. 2011. Changes in Rubisco kinetics during the evolution of C_4_ photosynthesis in *Flaveria* (Asteraceae) are associated with positive selection on genes encoding the enzyme. Molecular Biology and Evolution 28, 1491–1503. 21172830 10.1093/molbev/msq335

[CIT0042] Keerberg O , Pärnik T, Ivanova H, Bassüner B, Bauwe H. 2014. C_2_ photosynthesis generates about 3-fold elevated leaf CO_2_ levels in the C_3_–C_4_ intermediate species *Flaveria pubescens*. Journal of Experimental Botany 65, 3649–3656. 24916069 10.1093/jxb/eru239 PMC4085972

[CIT0043] Khoshravesh R , Stata M, Busch FA, et al. 2019. The evolutionary origin of C_4_ photosynthesis in the grass subtribe Neurachninae. Plant Physiology 182, 566–583. 31611421 10.1104/pp.19.00925 PMC6945869

[CIT0044] Khoshravesh R , Stinson CR, Stata M, Busch FA, Sage RF, Ludwig M, Sage TL. 2016. C_3_–C_4_ intermediacy in grasses: organelle enrichment and distribution, glycine decarboxylase expression, and the rise of C_2_ photosynthesis. Journal of Experimental Botany 67, 3065–3078. 27073202 10.1093/jxb/erw150 PMC4867898

[CIT0045] Kopka J , Schauer N, Krueger S, et al. 2005. GMD@CSB.DB: the Golm Metabolome Database. Bioinformatics 21, 1635–1638. 15613389 10.1093/bioinformatics/bti236

[CIT0046] Ku MS , Wu J, Dai Z, Scott RA, Chu C, Edwards GE. 1991. Photosynthetic and photorespiratory characteristics of *Flaveria* species. Plant Physiology 96, 518–528. 16668217 10.1104/pp.96.2.518 PMC1080801

[CIT0047] Ku MSB , Monson RK, Littlejohn RO, Nakamoto H, Fisher DB, Edwards GE. 1983. Photosynthetic characteristics of C_3_–C_4_ intermediate *Flaveria* species - I. Leaf anatomy, photosynthetic responses to O_2_ and CO_2_ and activities of key enzymes in the C_3_ and C_4_ pathways. Plant Physiology 75, 993–996. 10.1104/pp.71.4.944 PMC1066148 16662933

[CIT0048] Kubien DS , Whitney SM, Moore PV, Jesson LK. 2008. The biochemistry of Rubisco in *Flaveria*. Journal of Experimental Botany 59, 1767–1777. 18227079 10.1093/jxb/erm283

[CIT0049] Laetsch WM , Price I. 1969. Development of the dimorphic chloroplasts of sugar cane. American Journal of Botany 56, 77–87.

[CIT0050] Langdale JA. 2011. C_4_ cycles: past, present, and future research on C_4_ photosynthesis. The Plant Cell 23, 3879–3892. 22128120 10.1105/tpc.111.092098 PMC3246324

[CIT0051] Lawson T , Kramer DM, Raines CA. 2012. Improving yield by exploiting mechanisms underlying natural variation of photosynthesis. Current Opinion in Biotechnology 23, 215–220. 22296828 10.1016/j.copbio.2011.12.012

[CIT0052] Leegood RC. 1985. The intercellular compartmentation of metabolites in leaves of *Zea mays* L. Planta 164, 163–171. 24249557 10.1007/BF00396078

[CIT0053] Leegood RC , von Caemmerer S. 1994. Regulation of photosynthetic carbon assimilation in leaves of C_3_–C_4_ intermediate species of *Moricandia* and *Flaveria*. Planta 192, 232–238.

[CIT0054] Levey M , Timm S, Mettler-Altmann T, Borghi GL, Koczor M, Arrivault S, Weber AP, Bauwe H, Gowik U, Westhoff P. 2019. Efficient 2-phosphoglycolate degradation is required to maintain carbon assimilation and allocation in the C_4_ plant *Flaveria bidentis*. Journal of Experimental Botany 70, 575–587. 30357386 10.1093/jxb/ery370 PMC6322630

[CIT0055] Lisec J , Schauer N, Kopka J, Willmitzer L, Fernie AR. 2006. Gas chromatography mass spectrometry-based metabolite profiling in plants. Nature Protocols 1, 387–396. 17406261 10.1038/nprot.2006.59

[CIT0056] Lorimer GH , Andrews TJ. 1973. Plant photorespiration—an inevitable consequence of the existence of atmospheric oxygen. Nature 243, 359–360.

[CIT0057] Lundgren MR. 2020. C_2_ photosynthesis: a promising route towards crop improvement? New Phytologist 228, 1734–1740. 10.1111/nph.16494 32080851

[CIT0058] Lundgren MR , Osborne CP, Christin PA. 2014. Deconstructing Kranz anatomy to understand C_4_ evolution. Journal of Experimental Botany 65, 3357–3369. 24799561 10.1093/jxb/eru186

[CIT0059] Lyu MJA , Gowik U, Kelly S, et al. 2015. RNA-Seq based phylogeny recapitulates previous phylogeny of the genus *Flaveria* (Asteraceae) with some modifications. BMC Ecology and Evolution 15, 116. 10.1186/s12862-015-0399-9 PMC4472175 26084484

[CIT0060] Ma F , Jazmin LJ, Young JD, Allen DK. 2014. Isotopically nonstationary ^13^C flux analysis of changes in *Arabidopsis thaliana* leaf metabolism due to high light acclimation. Proceedings of the National Academy of Sciences, USA 111, 16967–16972. 10.1073/pnas.1319485111 PMC4250135 25368168

[CIT0061] Mallmann J , Heckmann D, Bräutigam A, Lercher MJ, Weber AP, Westhoff P, Gowik U. 2014. The role of photorespiration during the evolution of C_4_ photosynthesis in the genus *Flaveria*. eLife 3, e02478. 24935935 10.7554/eLife.02478 PMC4103682

[CIT0062] Matsuoka M , Furbank RT, Fukayama H, Miyao M. 2001. Molecular engineering of C_4_ photosynthesis. Annual Review of Plant Physiology and Plant Molecular Biology 52, 297–314. 10.1146/annurev.arplant.52.1.297 11337400

[CIT0063] McKown AD , Moncalvo JM, Dengler NG. 2005. Phylogeny of *Flaveria* (Asteraceae) and inference of C_4_ photosynthesis evolution. American Journal of Botany 92, 1911–1928. 21646108 10.3732/ajb.92.11.1911

[CIT0064] Meister M , Agostino A, Hatch MD. 1996. The roles of malate and aspartate in C_4_ photosynthetic metabolism of *Flaveria bidentis* (L). Planta 199, 262–269.

[CIT0065] Merlo L , Geigenberger P, Hajirezaei M, Stitt M. 1993. Changes of carbohydrates, metabolites and enzyme activities in potato tubers during development, and within a single tuber along a stolon–apex gradient. Journal of Plant Physiology 142, 392–402.

[CIT0066] Miyao M , Masumoto C, Miyazawa S, Fukayama H. 2011. Lessons from engineering a single-cell C_4_ photosynthetic pathway into rice. Journal of Experimental Botany 62, 3021–3029. 21459764 10.1093/jxb/err023

[CIT0067] Monson RK , Moore BD, Ku MS, Edwards GE. 1986. Co-function of C_3_- and C_4_-photosynthetic pathways in C_3_, C_4_ and C_3_–C_4_ intermediate *Flaveria* species. Planta 168, 493–502. 24232325 10.1007/BF00392268

[CIT0068] Monson RK , Rawsthorne S. 2000. CO_2_ assimilation in C_3_–C_4_ intermediate plants. In: Leegood RC, Sharkey TD, von Caemmerer S, eds. Photosynthesis. Advances in photosynthesis and respiration. Dordrecht: Springer 9, 533–550.

[CIT0069] Moore BD , Ku MSB, Edwards GE. 1987. C_4_ photosynthesis and light-dependent accumulation of inorganic carbon in leaves of C_3_–C_4_ and C_4_*Flaveria* species. Australian Journal of Plant Physiology 14, 657–68.

[CIT0070] Moore B , Ku MSB, Edwards GE. 1989. Expression of C_4_‐like photosynthesis in several species of *Flaveria*. Plant, Cell & Environment 12, 541–549.

[CIT0071] Muhaidat R , Sage RF, Dengler NG. 2007. Diversity of Kranz anatomy and biochemistry in C_4_ eudicots. American Journal of Botany 94, 362–381. 21636407 10.3732/ajb.94.3.362

[CIT0072] Muhaidat R , Sage TL, Frohlich MW, Dengler NG, Sage RF. 2011. Characterization of C₃–C₄ intermediate species in the genus *Heliotropium* L. (Boraginaceae): anatomy, ultrastructure and enzyme activity. Plant, Cell & Environment 34, 1723–1736. 10.1111/j.1365-3040.2011.02367.x 21631534

[CIT0073] Munekage YN. 2016. Light harvesting and chloroplast electron transport in NADP-malic enzyme type C_4_ plants. Current Opinion in Plant Biology 31, 9–15. 26999307 10.1016/j.pbi.2016.03.001

[CIT0074] Nakamura N , Iwano M, Havaux M, Yokota A, Munekage YN. 2013. Promotion of cyclic electron transport around photosystem I during the evolution of NADP-malic enzyme-type C_4_ photosynthesis in the genus *Flaveria*. New Phytologist 199, 832–842. 10.1111/nph.12296 23627567

[CIT0075] Osmond CB , Harris B. 1971. Photorespiration during C_4_ photosynthesis. Biochimica et Biophysica Acta 234, 270–282. 4327796 10.1016/0005-2728(71)90082-x

[CIT0076] Rawsthorne S , Hylton CM. 1991. The relationship between the post-illumination CO_2_ burst and glycine metabolism in leaves of C_3_ and C_3_–C_4_ intermediate species of *Moricandia*. Planta 186, 122–126. 24186584 10.1007/BF00201507

[CIT0077] Rawsthorne S , Hylton CM, Smith AM, Woolhouse HW. 1988a. Photorespiratory metabolism and immunogold localization of photorespiratory enzymes in leaves of C_3_ and C_3_–C_4_ intermediate species of *Moricandia*. Planta 173, 298–308. 24226536 10.1007/BF00401016

[CIT0078] Rawsthorne S , Hylton CM, Smith AM, Woolhouse HW. 1988b. Distribution of photorespiratory enzymes between bundle-sheath and mesophyll cells in leaves of the C_3_–C_4_ intermediate species *Moricandia arvensis* (L.) DC. Planta 176, 527–532. 24220949 10.1007/BF00397660

[CIT0079] Sage RF. 2002. Variation in the *k*_cat_ of Rubisco in C_3_ and C_4_ plants and some implications for photosynthetic performance at high and low temperature. Journal of Experimental Botany 53, 609–620. 11886880 10.1093/jexbot/53.369.609

[CIT0080] Sage RF. 2004. The evolution of C_4_ photosynthesis. New Phytologist 161, 341–370. 10.1111/j.1469-8137.2004.00974.x 33873498

[CIT0081] Sage RF. 2016. A portrait of the C_4_ photosynthetic family on the 50th anniversary of its discovery: species number, evolutionary lineages, and Hall of Fame. Journal of Experimental Botany 67, 4039–4056. 27053721 10.1093/jxb/erw156

[CIT0082] Sage RF , Christin PA, Edwards EJ. 2011. The C_4_ plant lineages of planet Earth. Journal of Experimental Botany 62, 3155–3169. 21414957 10.1093/jxb/err048

[CIT0083] Sage RF , Monson RK, Ehleringer JR, Adachi S, Pearcy RW. 2018. Some like it hot: the physiological ecology of C_4_ plant evolution. Oecologia 187, 941–966. 29955992 10.1007/s00442-018-4191-6

[CIT0084] Sage RF , Sage TL, Kocacinar F. 2012. Photorespiration and the evolution of C_4_ photosynthesis. Annual Review of Plant Biology 63, 19–47. 10.1146/annurev-arplant-042811-105511 22404472

[CIT0085] Sage TL , Busch FA, Johnson DC, Friesen PC, Stinson CR, Stata M, Sultmanis S, Rahman BA, Rawsthorne S, Sage RF. 2013. Initial events during the evolution of C_4_ photosynthesis in C_3_ species of Flaveria. Plant Physiology 163, 1266–1276. 24064930 10.1104/pp.113.221119 PMC3813649

[CIT0086] Salvucci ME. 1989. Regulation of Rubisco activity in vivo. Plant Physiology 77, 164–171.

[CIT0087] Schlüter U , Bräutigam A, Droz JM, Schwender J, Weber APM. 2018. The role of alanine and aspartate aminotransferases in C_4_ photosynthesis. Plant Biology 21, 64–76. 10.1111/plb.12904 30126035

[CIT0088] Schlüter U , Weber AP. 2016. The road to C_4_ photosynthesis: evolution of a complex trait via intermediary states. Plant & Cell Physiology 57, 881–889. 26893471 10.1093/pcp/pcw009

[CIT0089] Schlüter U , Weber APM. 2020. Regulation and evolution of C_4_ photosynthesis. Annual Review of Plant Biology 71, 183–215. 10.1146/annurev-arplant-042916-040915 32131603

[CIT0090] Schmitt MR , Edwards GE. 1981. Photosynthetic capacity and nitrogen use efficiency of maize, wheat, and rice: a comparison between C_3_ and C_4_ photosynthesis. Journal of Experimental Botany 32, 459–466.

[CIT0091] Schulze S , Mallmann J, Burscheidt J, Koczor M, Streubel M, Bauwe H, Gowik U, Westhoff P. 2013. Evolution of C_4_ photosynthesis in the genus *Flaveria*: establishment of a photorespiratory CO_2_ pump. The Plant Cell 25, 2522–2535. 23847152 10.1105/tpc.113.114520 PMC3753380

[CIT0092] Schüssler C , Freitag H, Koteyeva N, Schmidt D, Edwards G, Voznesenskaya E, Kadereit G. 2017. Molecular physlogeny and forms of photosynthesis in tribe Salsoleae (Chenopodiaceae). Journal of Experimental Botany 68, 207–223. 28003310 10.1093/jxb/erw432 PMC5853613

[CIT0093] Sharkey TD , Preiser AL, Weraduwage SM, Gog L. 2020. Source of ^12^C in Calvin Benson cycle intermediates and isoprene emitted from plant leaves fed with ^13^CO_2_. The Biochemical Journal 477, 3237–3252. 32815532 10.1042/BCJ20200480 PMC7666771

[CIT0094] Simkin AJ , López-Calcagno PE, Raines CA. 2019. Feeding the world: improving photosynthetic efficiency for sustainable crop production. Journal of Experimental Botany 70, 1119–1140. 30772919 10.1093/jxb/ery445 PMC6395887

[CIT0095] Stitt M , Heldt HW. 1985. Generation and maintenance of concentration gradients between the mesophyll and bundle sheath in maize leaves. Biochimica et Biophysica Acta 80, 400–414.

[CIT0096] Szecowka M , Heise R, Tohge T, et al. 2013. Metabolic fluxes in an illuminated Arabidopsis rosette. The Plant Cell 25, 694–714. 23444331 10.1105/tpc.112.106989 PMC3608787

[CIT0097] Usuda H , Edwards GE. 1980. Localization of glycerate kinase and some enzymes for sucrose synthesis in C_3_ and C_4_ plants. Plant Physiology 65, 1017–1022. 16661277 10.1104/pp.65.5.1017 PMC440468

[CIT0098] Weber AP , von Caemmerer S. 2010. Plastid transport and metabolism of C_3_ and C_4_ plants—comparative analysis and possible biotechnological exploitation. Current Opinion in Plant Biology 13, 257–265. 20188622 10.1016/j.pbi.2010.01.007

[CIT0099] Weissmann S , Ma F, Furuyama K, Gierse J, Berg H, Shao Y, Taniguchi M, Allen DK, Brutnell TP. 2016. Interactions of C_4_ subtype metabolic activities and transport in maize are revealed through the characterization of DCT2 mutants. The Plant Cell 28, 466–484. 26813621 10.1105/tpc.15.00497 PMC4790864

[CIT0100] Westhoff P , Gowik U. 2004. Evolution of C_4_ phosphoenolpyruvate carboxylase. Genes and proteins: a case study with the genus *Flaveria*. Annals of Botany 93, 13–23. 14644912 10.1093/aob/mch003 PMC4242257

[CIT0101] Williams BP , Johnston IG, Covshoff S, Hibberd JM. 2013. Phenotypic landscape inference reveals multiple evolutionary paths to C_4_ photosynthesis. eLife 2, e00961. 24082995 10.7554/eLife.00961 PMC3786385

[CIT0102] Woo KC , Anderson JM, Boardman NK, Downton WJS, Osmond CB, Thorne SW. 1970. Deficient photosystem II in agranal bundle sheath chloroplasts of C_4_ plants. Proceedings of the National Academy of Sciences, USA 67, 18–25. 10.1073/pnas.67.1.18 PMC283159 16591853

[CIT0103] Zachos JC , Dickens GR, Zeebe RE. 2008. An early Cenozoic perspective on greenhouse warming and carbon-cycle dynamics. Nature 451, 279–283. 18202643 10.1038/nature06588

