# Supplementary material for: Metabolic profiles in C3, C3–C4 intermediate, C4-like, and C4 species in the genus Flaveria
Source: J Exp Bot. 2021 Dec 15;73(5):1581–601. doi: 10.1093/jxb/erab540 (PMC8890617; doi:10.1093/jxb/erab540)
Supplement: erab540_suppl_supplementary_figures_S1-S4_tables_S2-S5 [file erab540_suppl_supplementary_figures_s1-s4_tables_s2-s5.pdf]

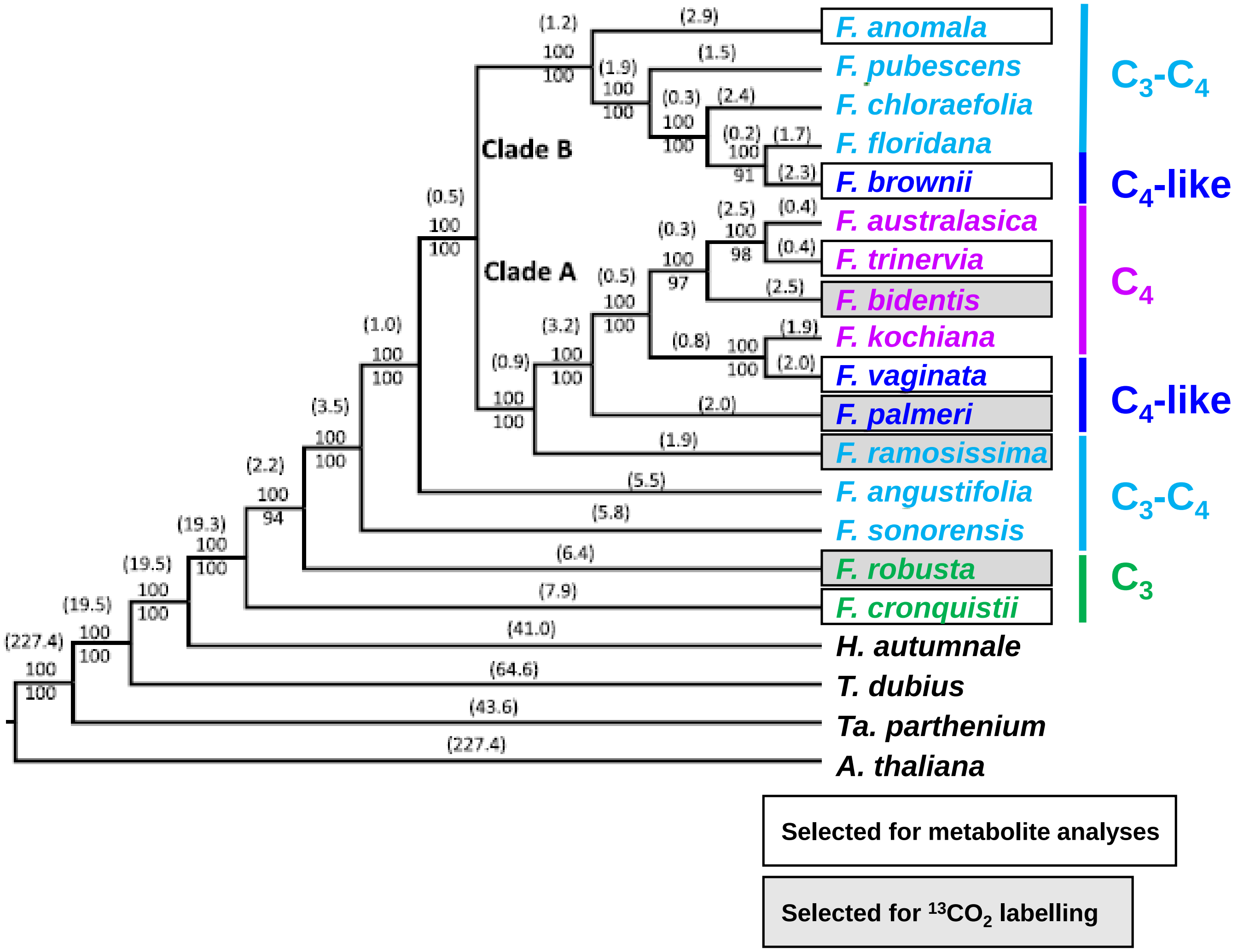

Supplementary Fig. S1. Phylogenetic tree of 16 *Flaveria* species. The figure, modified from Lyu *et al.* (2015), shows the phylogenetic tree of 16 *Flaveria* species together with three out-group Asteraceae species: *Helenium autumnale*, *Tragopogon dubius* and *Tanacetum parthenium. Arabidopsis thaliana* was included as a non-Asteraceae species. The phylogenetic tree was reconstructed using *m-CDS* sequences. For further details about the tree, refer to the original paper from Lyu *et al.* (2015). *Flaveria* species name are colored according to the photosynthetic mode, indicated on the right side of the figure. *Flaveria* species utilized in this paper for metabolite analyses and $^{13}CO_2$ labelling are highlighted according to the legend below the tree.

Legend is continued on the next page.

Supplementary Fig. S1. Phylogenetic tree of 16 *Flaveria* species. Continued.

Flaveria is a relatively young genus (2-3 Mya; Sage, 2016) belonging to the Asteraceae family, which finds its geographical origin in the Mesoamerican region (Powell, 1978). The genus include 23 species, comprising both C_3 and NADP-dependent malic enzyme (NADP-ME) subtype C_4 species, as well as many intermediates between these two photosynthetic modes (Apel and Maass, 1981; Ku *et al.,* 1983; Nakamoto *et al.,* 1983; Sudderth *et al.,* 2007). The phylogenetic tree comprises three C_3 species at the base of the *Flaveria* cluster, and then a division into two major clades: Clade A includes all of the complete C_4 species as well as several advanced intermediates, while Clade B includes C_3-C_4 intermediates, and one species (*F. brownii*) that has reached the status of C_4-like (Kopriva *et al.,* 1996; McKown *et al.,* 2005; Lyu *et al.,* 2015). Ancestral C_3 *Flaveria* species already possessed pre-conditioning elements that helped this genus proceed down the path of C_4 evolution, like the duplication of the gene encoding glycine decarboxylase P protein and the reduction in expression of one of these isoforms in mesophyll cells (Schulze et al., 2013, 2016). Furthermore, the attainment of the status of proto-Kranz (see *F. robusta* and F. *pringlei*; Sage *et al.,* 2012; Sage *et al.,* 2013; Sage *et al.,* 2014) favored the emergence of C_2 photosynthesis in ancestors of both clades: this idea is also strengthened by the presence at the tree base of *F. sonorensis*, a primitive C_3-C_4 intermediate (Moore *et al.,* 1987). Elements of the C_4 pathway were already present in early C_3-C_4 *Flaveria* intermediates like *F. anomala*; however, not as carbon assimilation-relevant as in advanced C_3-C_4 species like *F. ramosissima*, which assimilate around 50% of their carbon through PEPC (Moore *et al.,* 1987; Chastain and Chollet, 1989). Apart from *F. brownii*, which has an ancestral C_4-like phenotype (Monson *et al.,* 1987; Cheng *et al.,* 1988), all the other C_4-like *Flaveria* species are more advanced and fix >90% of their carbon using a C_4 cycle (Moore *et al.,* 1989).

For references see Literature list in the main manuscript plus the following:

Apel P, Maass I. 1981. Photosynthesis in Species of Flaveria: CO_2 Compensation Concentration, O_2 Influence on Photosynthetic Gas Exchange and $^{\delta 13}$C Values in Species of Flaveria (Asteraceae). Biochemie und Physiologie der Pflanzen 176, 396-399.

Kopriva S, Chu CC, Bauwe H. 1996. Molecular phylogeny of Flaveria as deduced from the analysis of nucleotide sequences encoding the H-protein of the glycine cleavage system. Plant, Cell & Environment 19, 1028-1036.

Monson RK, Schuster WS, Ku MSB. 1987. Photosynthesis in *Flaveria brownii* AM Powell: A C_4-Like C_3-C_4 Intermediate. Plant Physiology 85, 1063-1067.

Nakamoto H, Ku MSB, Edwards GE. 1983. Photosynthetic characteristics of C_3-C_4 intermediate flaveria species II. Kinetic properties of phosphoenolpyruvate carboxylase from C_3, C_4 and C_3-C_4 intermediate species. Plant and Cell Physiology 24, 1387-1393.

Powell AM. 1978. Systematics of Flaveria (Flaveriinae-Asteraceae). Annals of the Missouri Botanical Garden 65, 590-636.

Sage RF, Khoshravesh R, Sage TL. 2014. From proto-Kranz to C_4 Kranz: Building the bridge to C_4 photosynthesis. Journal of Experimental Botany 65, 3341-3356.

Schulze S, Westhoff P, Gowik U. 2016. Glycine decarboxylase in C_3, C_4 and C_3-C_4 intermediate species. Current Opinion in Plant Biology 31, 29-35.

Sudderth EA, Muhaidat RM, McKown AD, Kocacinar F, Sage RF. 2007. Leaf anatomy, gas exchange and photosynthetic enzyme activity in *Flaveria kochiana*. Functional Plant Biology 34, 118-129.

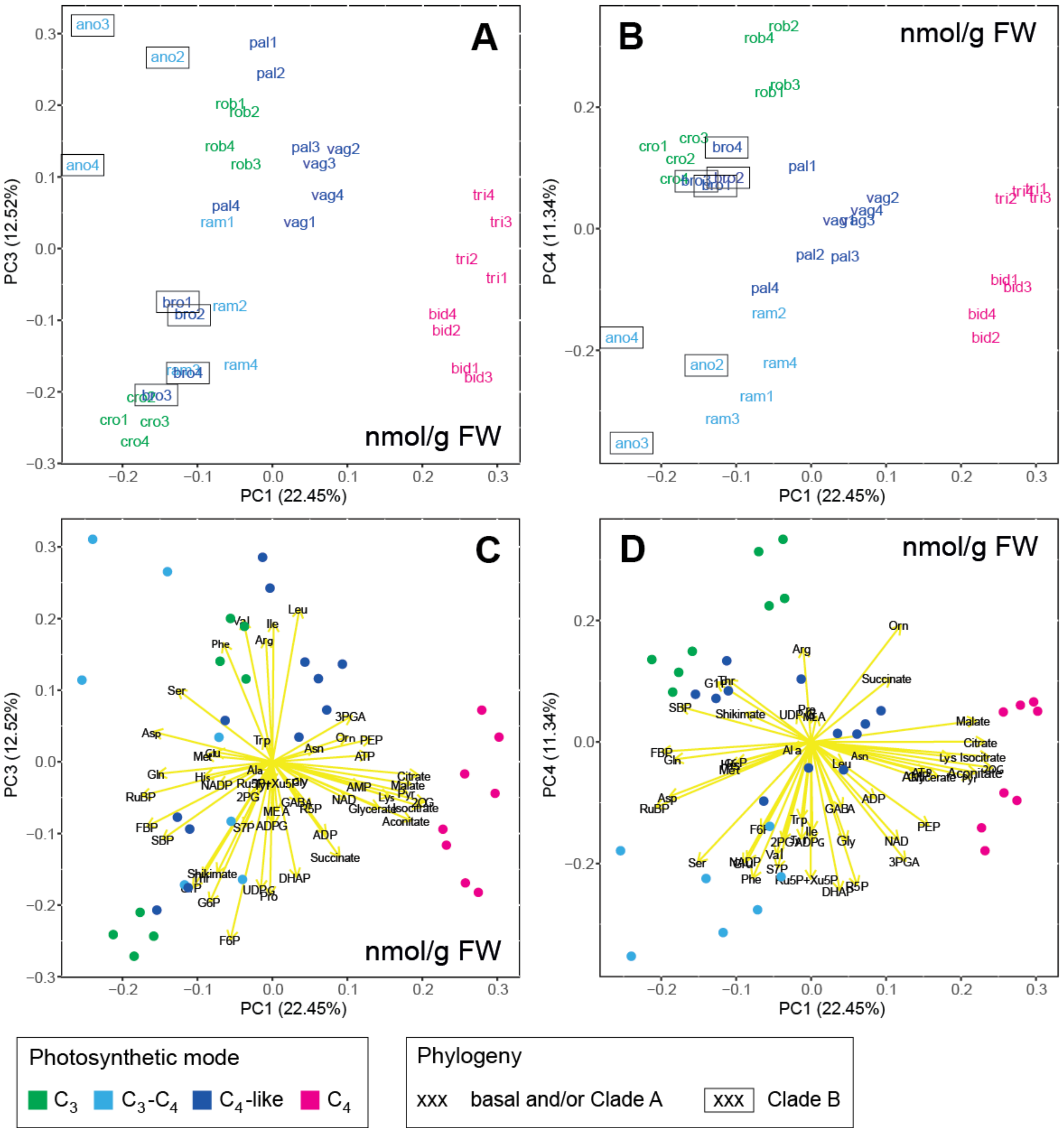

Supplementary Fig. S2. Principal component (PC) analyses of metabolite profiles in nine *Flaveria* species: PC1 in combination with PC3 or PC4. This figure is supplementary to Fig. 2. Distribution of samples along PC1 and either PC3 **(A)** or PC4 **(B)**, with each sample being represented by a colored label indicating the species and biological replicate number. **(C, D)** Metabolite eigenvectors driving sample separation are shown in yellow, while individual samples appear as colored dots. In all panels, the color code represents the different photosynthetic modes, as indicated by the legend below the figure. In panels **A** and **C**, a box denotes Clade B species, as indicated by the legend below the figure. Species abbreviations are, alphabetically: ano, *F. anomala* (Clade B, C_3-C_4); bid, *F. bidentis* (Clade A, C_4); bro, *F. brownii* (Clade B, C_4-like); cro, *F. cronquistii* (basal, C_3); pal, *F. palmeri* (Clade A, C_4-like); ram, *F. ramosissima* (Clade A, C_3-C_4); rob, *F. robusta* (basal, C_3) ; tri, *F. trinervia* (Clade A, C_4); vag, *F. vaginata* (Clade A, C_4-like). For metabolite abbreviations refer to Supplementary Table S2, and for the original data see Supplementary Dataset S1.

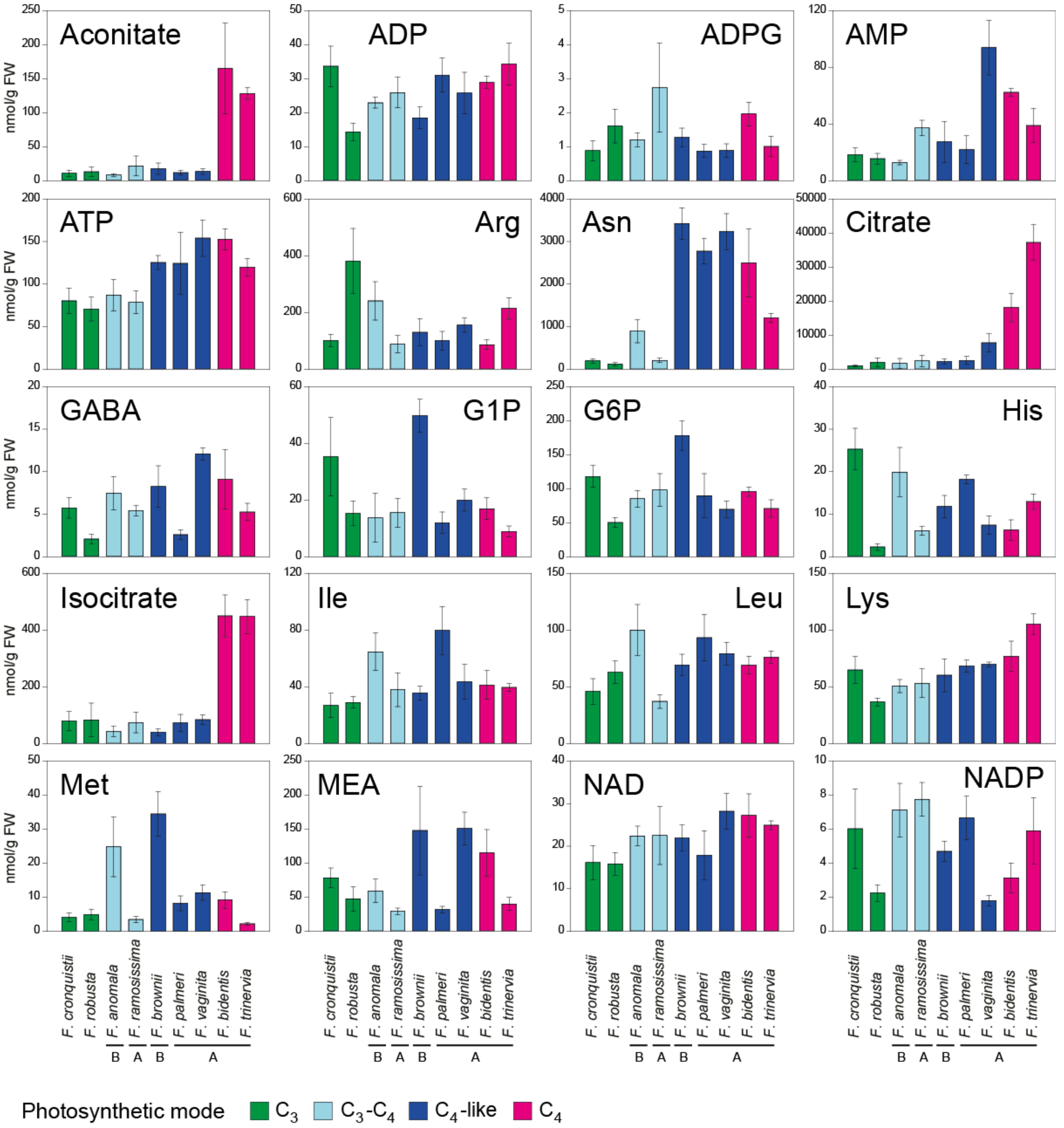

Supplementary Fig. S3. Absolute amounts of additional metabolites in nine *Flaveria* species. This figure is supplementary to Fig. 3. Metabolites are ordered alphabetically, from top to bottom. The color of each bar represent the different photosynthetic modes, as indicated by the legend below the figure. In the sets of C_3-C_4 and C_4-like species, the Clade B species (*F. anomala* and *F. brownii,* respectively) are placed to the left of the Clade A species; clade is also indicated as 'A' or 'B' below the species name. The amounts are plotted as average (nmol/g FW) ± SD (n=3 to 4). Letters above each bar represent post-hoc pairwise comparison grouping (Holm-Sidak method, p<0.05). For metabolite abbreviations, refer to Supplementary Table S2, and for the original data see Supplementary Dataset S1. The data in **(B)** for CBC metabolite levels in the two complete C_4 species *F. bidentis* and *F. trinervia* were included in a previous publication (Arrivault *et al.*, 2019). This figure is continued on the next page.

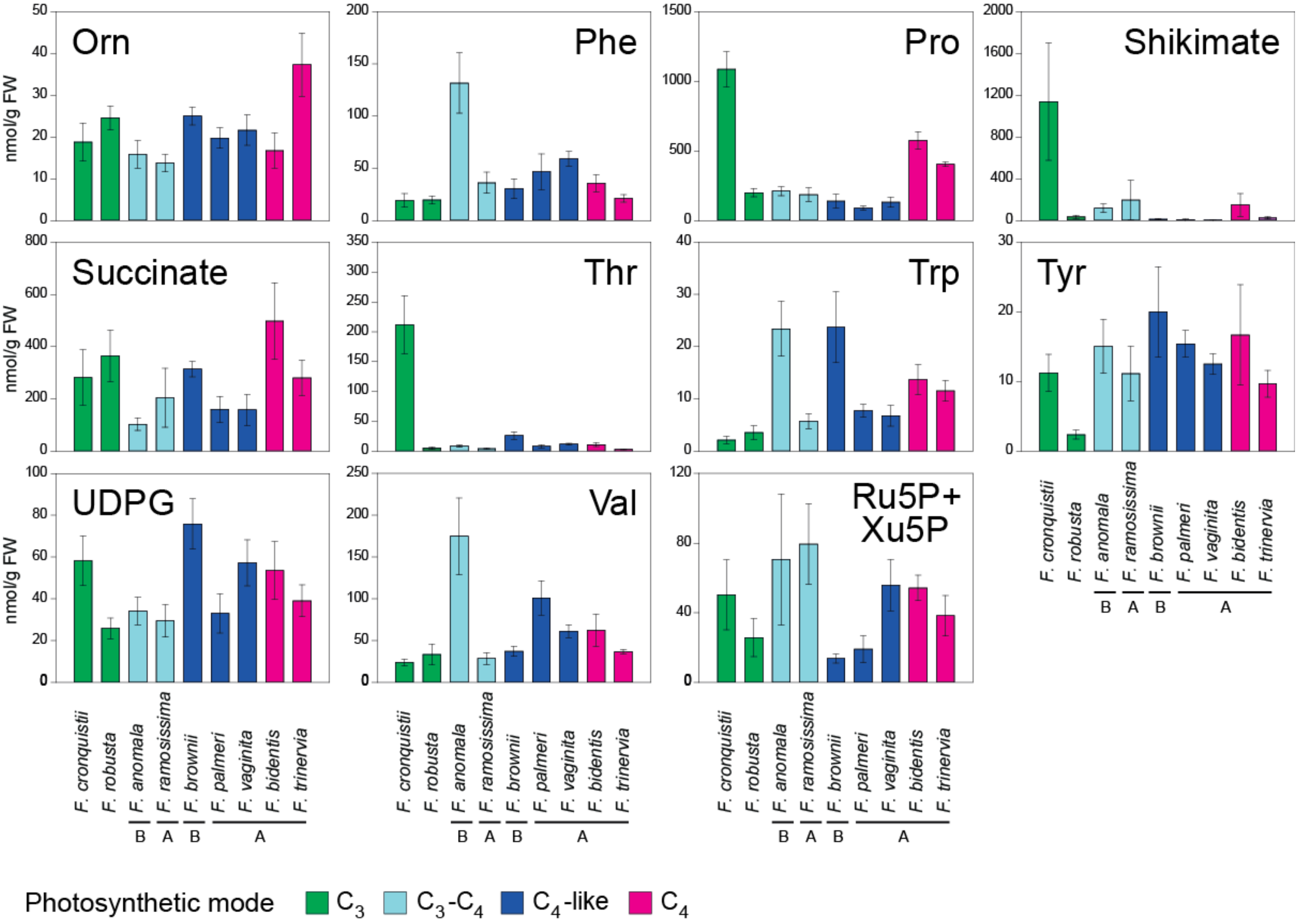

Supplementary Fig. S3. Absolute amounts of additional metabolites in nine *Flaveria* species.
Continued.

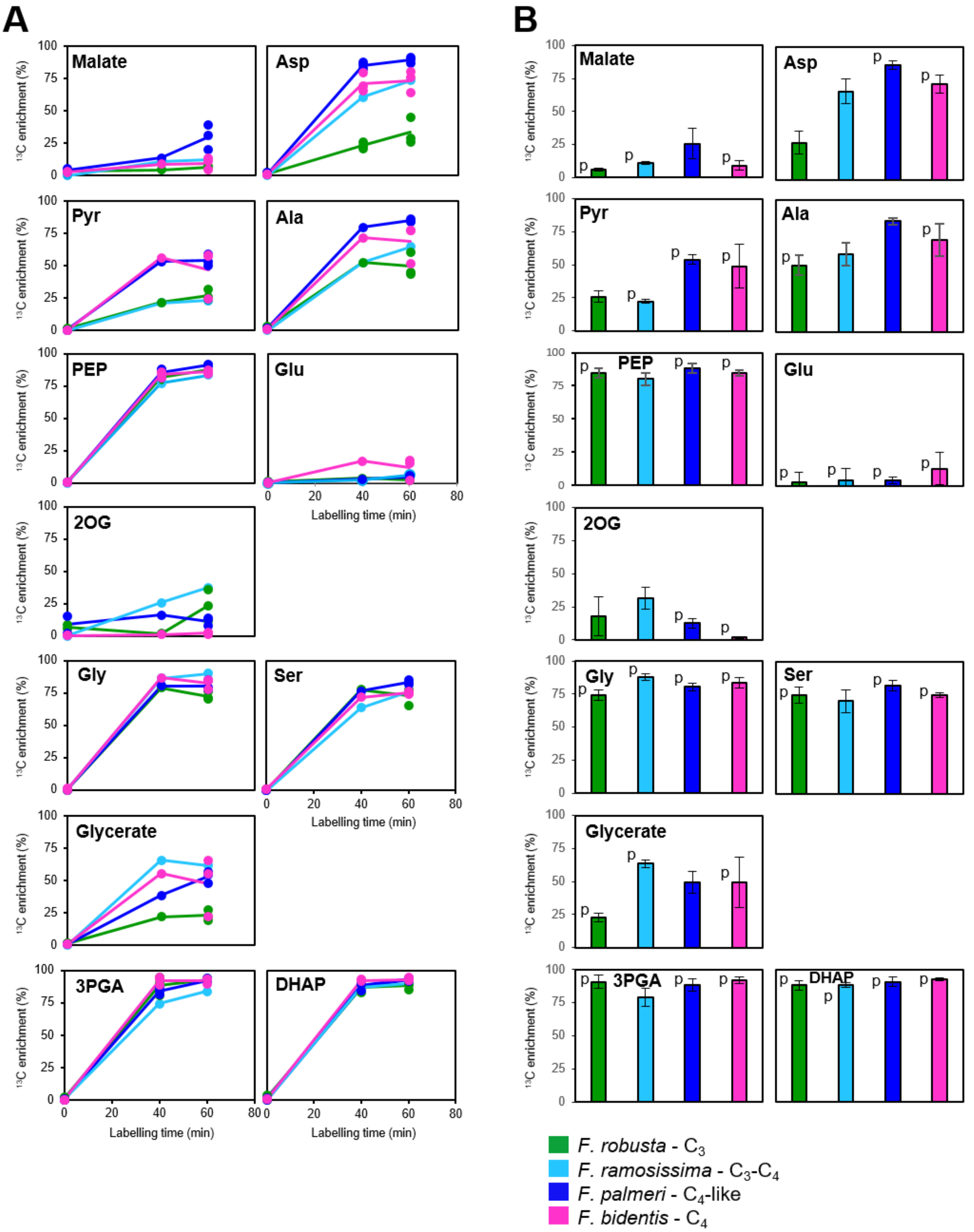

Supplementary Fig. S4. ^{13}C enrichment in key metabolites in four Clade A *Flaveria* species with different modes of photosynthesis. This figure is supplementary to Fig. 5. **(A)** Time course of ^{13}C enrichment (%). Each point represents a single sample, while the line represents the average trend. **(B)** ^{13}C enrichment (%) after 40-60 min. The figure represents a summary of the data shown in **(A)**. Values at 40 and 60 min were combined to calculate average enrichment ± SD. The density of sampling was restricted by availability of plant material, with n=2 samples for 0 min, n=1 for 40 min and n=3 for 60 min for GC-TOF-MS analysis and n=3 samples for 0, 40 and 60 min for LC-MS/MS analysis. For *F. ramosissima*, only 40 and 60 min pulses were performed (both n=1). The letter "p" indicates if the ^{13}C enrichment reached a plateau, defined as the average of the 60 min time points being the same or lower than the 40 min time point.The color of the dot and line **(A)** or bar **(B)** represent the different photosynthetic modes, as indicated by the legend in panel **B**. For metabolite abbreviations, refer to Supplementary Table S2, and for the original data, see Supplementary Dataset S2.

Metabolite	Abbreviation	Analytical method
2-oxoglutarate	2OG	LC-MS/MS + GC-TOF-MS for ^{13}C enrichment
2-phosphoglycolate	2PG	LC-MS/MS
3-phosphoglycerate	3PGA	Enzymatic (amount) + LC-MS/MS for ^{13}C enrichment
Aconitate	-	LC-MS/MS
Adenosine diphosphate	ADP	LC-MS/MS
Adenosine diphosphate glucose	ADPG	LC-MS/MS
Adenosine monophosphate	AMP	LC-MS/MS
Adenosine triphosphate	ATP	Enzymatic
Alanine	Ala	HPLC + GC-TOF-MS for ^{13}C enrichment
Arginine	Arg	HPLC
Asparagine	Asn	HPLC
Aspartate	Asp	LC-MS/MS (also for ^{13}C enrichment)
Citrate	-	LC-MS/MS
Dihydroxyacetone phosphate	DHAP	LC-MS/MS (also for ^{13}C enrichment)
Fructose-6-phosphate	F6P	LC-MS/MS
Fructose-1,6-bisphosphate	FBP	LC-MS/MS
γ-aminobutyric acid	GABA	HPLC
Glucose-1-phosphate	G1P	LC-MS/MS
Glucose-6-phosphate	G6P	LC-MS/MS
Glutamine	Gln	HPLC
Glutamate	Glu	LC-MS/MS + GC-TOF-MS for ^{13}C enrichment
Glycerate	-	LC-MS/MS + GC-TOF-MS for ^{13}C enrichment
Glycine	Gly	HPLC + GC-TOF-MS for ^{13}C enrichment
Histidine	His	HPLC
Isocitrate	-	LC-MS/MS
Isoleucine	Ile	HPLC

Supplementary Table S2. List of measured metabolites, their abbreviations and analytical methods used. Indicated in red are the analytical methods used to calculate ^{13}C enrichments after $^{13}CO_2$ labelling experiments. More details on single analytical techniques can be found in the following papers: LC-MS/MS (Arrivault *et al.*, 2009, 2015, 2017), GC-TOF-MS (Lisec *et al.*, 2006; Huege *et al.*, 2014), HPLC (Carillo *et al.*, 2005) and enzymatic (Merlo *et al.*, 1993). The table is continued on the next page.

Metabolite	Abbreviation	Analytical method
Leucine	Leu	HPLC
Lysine	Lys	HPLC
Malate	-	LC-MS/MS+ GC-TOF-MS for ^{13}C enrichment
Methionine	Met	HPLC
Monoethanolamine	MEA	HPLC
Nicotinamide adenine dinucleotide	NAD	LC-MS/MS
Nicotinamide adenine dinucleotide phosphate	NADP	LC-MS/MS
Ornithine	Orn	HPLC
Phospho*enol*pyruvate	PEP	Enzymatic (amount) + LC-MS/MS for ^{13}C enrichment
Phenylalanine	Phe	HPLC
Proline	Pro	Enzymatic
Pyruvate	Pyr	Enzymatic (amount) + GC-TOF-MS for ^{13}C enrichment
Ribose 5-phosphate	R5P	LC-MS/MS
Ribulose 5-phosphate	Ru5P	LC-MS/MS
Ribulose 1,5-bisphosphate	RuBP	LC-MS/MS
Sedoheptulose 7-phosphate	S7P	LC-MS/MS
Sedoheptulose 1,7-phosphate	SBP	LC-MS/MS
Serine	Ser	HPLC+ GC-TOF-MS for ^{13}C enrichment
Shikimate	-	LC-MS/MS
Succinate	-	LC-MS/MS
Threonine	Thr	HPLC
Tryptophan	Trp	HPLC
Tyrosine	Tyr	HPLC
Uridine diphosphate glucose	UDPG	LC-MS/MS
Valine	Val	HPLC
Xylulose 5-phosphate	Xu5P	LC-MS/MS

Supplementary Table S2. List of measured metabolites, their abbreviations and analytical methods used.
Continued

Principal component	Standard Deviation	Proportion of variance (%)	Cumularive Proportion (%)
**PC1**	3.38	22.45	22.45
**PC2**	2.91	16.66	39.11
**PC3**	2.53	12.52	51.63
**PC4**	2.40	11.34	62.97
**PC5**	2.09	8.54	71.50
**PC6**	1.79	6.29	77.79
**PC7**	1.60	5.03	82.82
**PC8**	1.32	3.44	86.26
**PC9**	1.06	2.20	88.46
**PC10**	0.96	1.80	90.26

All metabolites
Nine *Flaveria* species
nmol/g FW

**PC1**	4.61	41.68	41.68
**PC2**	2.68	14.10	55.77
**PC3**	2.37	11.01	66.78
**PC4**	1.83	6.53	73.32
**PC5**	1.73	5.87	79.19
**PC6**	1.69	5.57	84.76
**PC7**	1.38	3.71	88.47
**PC8**	1.03	2.08	90.55
**PC9**	0.89	1.56	92.11
**PC10**	0.84	1.38	93.49

All metabolites
Nine *Flaveria* species
Dimensionless

Supplementary Table S3. Summary of principal components for analyses on the entire *Flaveria* dataset. This table is supplementary to Fig. 2 and Supplementary Fig. S3. Summary of first ten principle components (PC) for PC analysis performed for nine *Flaveria* species using either FW-normalized or dimensionless datasets. Original data are available in Supplementary Dataset S1. For details of the dimensionless normalization see main text and Arrivault *et al*. (2019).

Flaveria robusta (C_3)

Metabolite	Absolute amount (nmol/g FW)	^{13}C enrichment (%)	Active pool (nmol/g FW)	Inactive pool (nmol/g FW)
Malate	1471	6	85	1386
Asp	876	21	183	693
Pyr	15	26	4	11
Ala	155	51	78	77
PEP	30	85	25	5
2OG	319	18	57	262
Glu	1914	3	54	1860
Gly	65	74	48	17
Ser	952	75	710	242
Glycerate	537	31	168	369
3PGA	135	91	123	12
DHAP	25	88	22	3

Flaveria ramosissima (C_3-C_4)

Metabolite	Absolute amount (nmol/g FW)	^{13}C enrichment (%)	Active pool (nmol/g FW)	Inactive pool (nmol/g FW)
Malate	1334	11	152	1182
Asp	1152	66	755	396
Pyr	32	22	7	25
Ala	200	59	118	83
PEP	60	80	48	12
2OG	410	32	130	280
Glu	3150	4	130	3019
Gly	108	88	96	13
Ser	1693	70	1187	506
Glycerate	757	65	489	268
3PGA	259	79	204	54
DHAP	53	89	47	6

Flaveria palmeri (C_4-like)

Metabolite	Absolute amount (nmol/g FW)	^{13}C enrichment (%)	Active pool (nmol/g FW)	Inactive pool (nmol/g FW)
Malate	4218	26	1088	3130
Asp	845	62	525	320
Pyr	165	54	89	76
Ala	1641	84	1373	268
PEP	106	88	93	12
2OG	1133	13	142	990
Glu	2698	4	111	2587
Gly	38	81	31	8
Ser	200	82	164	37
Glycerate	125	52	65	61
3PGA	294	88	260	34
DHAP	41	91	37	4

Flaveria bidentis (C_4)

Metabolite	Absolute amount (nmol/g FW)	^{13}C enrichment (%)	Active pool (nmol/g FW)	Inactive pool (nmol/g FW)
Malate	13632	9	1246	12386
Asp	360	23	81	279
Pyr	326	49	160	166
Ala	1297	70	903	395
PEP	124	85	106	19
2OG	8383	2	150	8233
Glu	2167	13	281	1886
Gly	78	83	65	13
Ser	171	75	128	43
Glycerate	866	52	452	415
3PGA	297	92	274	24
DHAP	60	93	55	4

Maize (C_4)

Metabolite	Absolute amount (nmol/g FW)	^{13}C enrichment (%)	Active pool (nmol/g FW)	Inactive pool (nmol/g FW)
Malate	4158	42	1762	2397
Asp	110	88	98	13
Pyr	153	76	116	37
Ala	1994	84	1675	319
PEP	101	92	93	8
2OG	639	17	107	532
Glu	2516	18	450	2066
Gly	145	77	112	33
Ser	291	68	197	94
Glycerate	436	26	114	322
3PGA	683	94	643	40
DHAP	328	94	308	20

Supplementary Table S4. Use of metabolite amounts and ^{13}C enrichments to estimate active and inactive pools in four *Flaveria* species and maize. This table is supplementary to Fig. 5 and summarizes absolute metabolite amounts (nmol/g FW), ^{13}C enrichment (%), active and inactive pools (nmol/g FW) in four *Flaveria* species and maize. Data for maize are from Arrivault *et al.* (2017) and additional 3PGA quantification. Metabolites shown are involved in the CCM, the photorespiratory pathway, and two metabolites from the CBC. For the *Flaveria* species, averaged amounts are from three to four biological replicates (see Supplementary Dataset S1), while the averaged ^{13}C enrichments are from combined 40 and 60 min labelling time points (n= 2 to 6; see Supplementary Dataset S2). Active pools were calculated as the amount multiplied by the fractional ^{13}C enrichment. The inactive pools were calculated by subtracting the active pool from the total amount. Note that for some species and in certain metabolites (malate, Asp, Pyr, Ala, 2OG and glycerate), a clear plateau was not reached (see Supplementary Fig. S4); consequently, the enrichment values in this table are only minimum approximations. For metabolite abbreviations, refer to Supplementary Table S2.

Principal component	Standard Deviation	Proportion of variance (%)	Cumularive Proportion (%)
**PC1**	1.74	30.43	30.43
**PC2**	1.56	24.48	54.91
**PC3**	1.17	13.75	68.65
**PC4**	1.05	11.11	79.77
**PC5**	0.91	8.34	88.11
**PC6**	0.75	5.62	93.73
**PC7**	0.65	4.26	97.98
**PC8**	0.33	1.10	99.09
**PC9**	0.30	0.92	100
**PC10**	0.00	0.0	100

Supplementary Table S5. Summary of first 10 PCs for PC analyses on the dimensionless multispecies dataset for CBC metabolites and 2PG. This table is supplementary to Fig. 6. The PC analysis was performed with CBC metabolites and 2PG for the nine *Flaveria* species plus five further C_3 species and two further C_4 species. The data for the additional species are from Arrivault *et al*. (2019). The table summarizes the fraction of total variance captured in the first ten PCs. The PC analysis was performed on a multispecies dimensionless dataset (for details see main text and Arrivault *et al*., 2019). Original data are available in Supplementary Dataset S3.
